# Unique Carbonate-Based Single Ion Conducting Block Copolymers Enabling High-Voltage, All-Solid-State Lithium Metal Batteries

**DOI:** 10.1021/acs.macromol.1c00981

**Published:** 2021-07-14

**Authors:** Gabriele Lingua, Patrick Grysan, Petr S. Vlasov, Pierre Verge, Alexander S. Shaplov, Claudio Gerbaldi

**Affiliations:** † GAME Lab, Department of Applied Science and Technology (DISAT), Politecnico di Torino, Corso Duca degli Abruzzi 24, Torino 10129, Italy; ‡ National Reference Center for Electrochemical Energy Storage (GISEL) - INSTM, Via G. Giusti 9, Firenze 50121, Italy; § Luxembourg Institute of Science and Technology (LIST), 5 Avenue des Hauts-Fourneaux, Esch-sur-Alzette L-4362, Luxembourg; ∥ Department of Macromolecular Chemistry, Saint-Petersburg State University, Universitetsky pr. 26, Saint Petersburg 198504, Russia

## Abstract

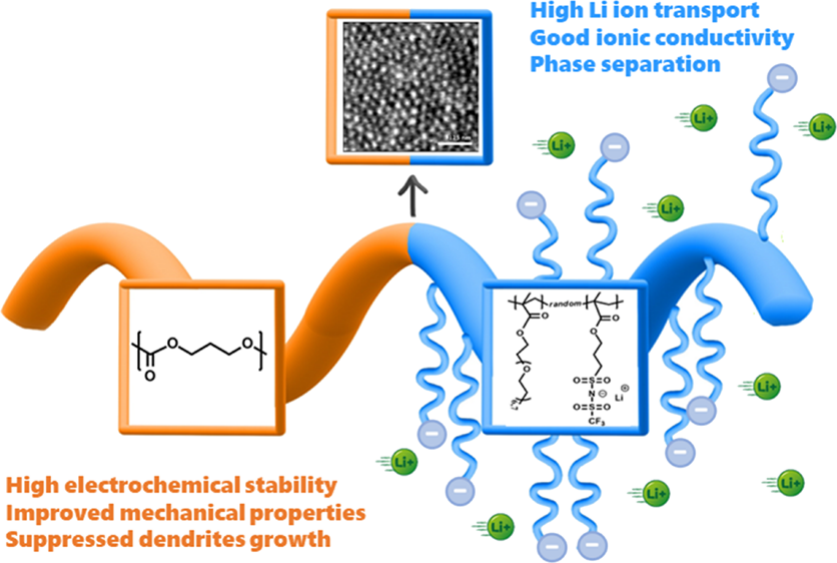

Safety and high-voltage operation are key metrics for advanced, solid-state energy storage devices to power low- or zero-emission HEV or EV vehicles. In this study, we propose the modification of single-ion conducting polyelectrolytes by designing novel block copolymers, which combine one block responsible for high ionic conductivity and the second block for improved mechanical properties and outstanding electrochemical stability. To synthesize such block copolymers, the ring opening polymerization (ROP) of trimethylene carbonate (TMC) monomer by the RAFT-agent having a terminal hydroxyl group is used. It allows for the preparation of a poly(carbonate) macro-RAFT precursor that is subsequently applied in RAFT copolymerization of lithium 1-[3-(methacryloyloxy)propylsulfonyl]-1-(trifluoromethylsulfonyl)imide and poly(ethylene glycol) methyl ether methacrylate. The resulting single-ion conducting block copolymers show improved viscoelastic properties, good thermal stability (*T*_onset_ up to 155 °C), sufficient ionic conductivity (up to 3.7 × 10^–6^ S cm^–1^ at 70 °C), and high lithium-ion transference number (0.91) to enable high power. Excellent plating/stripping ability with resistance to dendrite growth and outstanding electrochemical stability window (exceeding 4.8 V vs Li^+^/Li at 70 °C) are also achieved, along with enhanced compatibility with composite cathodes, both LiNiMnCoO_2_ – NMC and LiFePO_4_ – LFP, as well as the lithium metal anode. Lab-scale truly solid-state Li/LFP and Li/NMC lithium-metal cells assembled with the single-ion copolymer electrolyte demonstrate reversible and very stable cycling at 70 °C delivering high specific capacity (up to 145 and 118 mAh g^–1^, respectively, at a C/20 rate) and proper operation even at a higher current regime. Remarkably, the addition of a little amount of propylene carbonate (∼8 wt %) allows for stable, highly reversible cycling at a higher C-rate. These results represent an excellent achievement for a truly single-ion conducting solid-state polymer electrolyte, placing the obtained ionic block copolymers on top of polyelectrolytes with highest electrochemical stability and potentially enabling safe, practical Li-metal cells operating at high-voltage.

## Introduction

The global rechargeable battery market is forecasted to reach 250 billion euros per year by 2025, around 60% of which is committed for the advanced materials market and the rest is for investments in manufacturing capacity, R&D, and other supporting activities. The growth in electrification of modern society in the next decade will mainly be driven by the irreversible move toward decarbonization in many critical sectors, and batteries are identified as high-performance systems to significantly reduce the carbon footprint of the transportation sector, stabilize the power grid, and support a wide range of strategic industries.^1^ With such a rising demand across all application areas, there is an industrial pull to manufacture high-quality batteries providing enhanced energy/power densities, safety, and low-cost, along with a long cycle life and calendar lifetime.^2^

Lithium-ion batteries (LiBs) are key technology due to their high energy density, lightweight, fast charge/discharge, and long lifetime. Typical electrolytes for commercial LiBs are liquids or gels, soaked in a porous plastic layer, acting both as a storage reservoir and electronic separator of positive and negative electrodes. The two main advantages of liquid electrolytes are high ionic conductivity and excellent wetting properties of the active material particles that account for rather fast ionic diffusion in the bulk and low charge-transfer interfacial resistance. However, liquid electrolytes suffer from relatively high reactivity and thermodynamic instability at the electrode/electrolyte interface.^3,4^ This is even more problematic with lithium metal: its volumetric changes, induced by the formation of high-surface-area lithium, account for the generation of an unstable solid electrolyte interphase (SEI) and related dendrite growth, which affect the cycling performances.^5^ In addition, any voltage (overcharge) or temperature (overheating during high power use) fluctuations often correspond to cell instability and safety problems (fire breakout, gas evolution, depressurization of the battery case, etc.).^6,7^

Solid-state battery technology has recently gathered considerable attention from world-leading companies (e.g., Toyota, BMW, Dyson) and remains among the most promising solutions to power the next generation of electric vehicles including commercial and light duty vehicles, buses, and trucks due to the advantages in terms of energy density, safety, and processability.^8^ In the last decade, Blue Solutions has demonstrated the technical viability of the LMP battery in car sharing services, city buses, and stationary electrical storage. These demonstrations were particularly impressive in hot climates where LiB-based systems typically suffer from premature capacity fading.^9^

The scientific community is very active in finding the best truly solid-state polymer electrolyte (SPE).^10^ The task is still very challenging, and meeting safety and performance requirements is presently the key hurdle to be overcome to enable widespread commercialization of next-generation solid-state LiBs.^11−13^

Among SPEs, a new class of polyelectrolytes, namely, “single ion conducting polyelectrolytes” (SICPs), has deserved considerable attention.^14−20^ SICPs are composed of a polymer backbone bearing covalently bonded anionic functional groups along with free-to-move lithium counter ions responsible for cation mobility.^16^ Because of the single-ion nature, their lithium transference number values approach unity, with remarkable benefits to the electrochemical performance, as Li^+^ ions are predominantly engaged in the redox reaction while anions remain relatively inactive. The characteristics of such electrolyte materials in terms of ionic conductivity, Li^+^ ion transference number (*t*_Li_^+^), electrochemical stability, flexibility, and processability are strictly related to the type of polymer backbone, nature, and charge delocalization of anchored anions.^16^ Early reports on SICPs obtained via free radical polymerization were mainly focused on monomers bearing strongly coordinating anions, such as carboxylates^21^ and sulfonates.^22^ Further, the family of SICPs was expanded with polyelectrolytes bearing borate,^23−25^ phosphate,^26^ 1,2,3-triazolate,^27^ aluminate ([Al(OR)_4_]^−^), and thioaluminate ([Al(SR)_4_]^−^) anions.^28^ However, the real breakthrough in terms of the increase of ionic conductivity was achieved after the introduction of weakly coordinating and highly delocalized anions structurally similar to the well-known bis(trifluoromethylsulfonyl)imide (TFSI^−^) ion.^19,20,29−33^ The introduction of the TFSI or fluorosulfonylimide (FSI)-like^34^ anchored anions allowed a 2 to 3 order of magnitude increase in the ionic conductivity of SICPs in comparison with those bearing sulfonate and borate anions. Despite the rather weak coordination between the anions and the lithium cations, the glass transition temperatures (*T*_g_) of such homopolymers were still relatively high (>90 °C). Thus, the next improvement was attained either by the copolymerization of ionic monomers with neutral ones having long flexible oxyethylene segments, such as poly(ethylene glycol) methyl ether methacrylate (PEGM),^20,35^ or by the synthesis of triblock copolymers, growing the ionic blocks from central polyethylene oxide blocks with variable molar masses.^33,36^ Both these approaches allowed for lowering the *T*_g_ of SICPs and, thus, for enhancing their ionic conductivity to a higher level (up to 10^–6^ S cm^–1^ at 25 °C).

Apart from ionic conductivity and Li^+^ transference number, other fundamental features of SICPs include a wide electrochemical stability window and compatibility with the active materials, particularly lithium metal. The electrochemical stability defines the potential interval where the polyelectrolyte remains stable to the electrochemical reactions occurring at the interface with the electrodes. While the presence of ethylene oxide (EO) units tends to decrease the glass transition of SICPs and, consequently, to increase the ionic conductivity, it generally restricts the electrochemical stability of such polyelectrolytes vs Li^+^/Li in the range of 4.0–4.5 V.^20,35−37^ The promotion of high-voltage stability in poly(ethylene carbonate)/Li salt polymer electrolytes in comparison with PEO/Li salt systems was demonstrated previously.^38−40^ Mecerreyes and co-workers have recently applied the same approach to SICPs consisting of a combination of -EO- and carbonate units in a single polymer backbone.^41^ Such introduction of carbonate groups allowed an increase in electrochemical stability of SICPs up to 4.9 V vs Li^+^/Li at 70 °C.^41^

Several examples of solid-state SICP electrolytes delivering excellent results on the anode side have been already reported to date;^16^ conversely, the long-term stability with high-voltage cathodes (e.g., lithium nickel manganese cobalt oxide–NMC at low Co content or Li-rich NMC) is still compromised due to the limited electrochemical stability at anodic voltage values above 4.5 V vs Li^+^/Li. To the best of our knowledge, the only SICP successfully tested with a high voltage NMC-based cathode in a lab-scale cell was the abovementioned polyelectrolyte having the poly(ethylene oxide carbonate) main chain.^41^ Its single Li-ion conducting features along with high voltage stability resulted in good performances of NMC-based half cells at a C/20 rate and 70 °C in the 2.8–4.2 V range. Notwithstanding the promising cycling results in NMC-based half cells with SICP derived from poly(ethylene oxide carbonate),^41^ the Li metal anode required a special pretreatment with 2 M solution of lithium bis(fluorosulfonyl)imide (LiFSI) in dimethoxyethane. This was explained by the failure of the SICP electrolyte to form a highly conductive SEI layer under the defined cycling conditions. Such a limitation can be overcome by the design of an SICP macromolecular architecture that will lead to the formation of a Li metal/electrolyte interface in which carbonate units will form the predominant domain. Previously, it was shown that the synthesis of SICPs in the form of block copolymers may lead to the desired phase separation and even to an increase in ionic conductivity.^42−44^

**Scheme 1:**
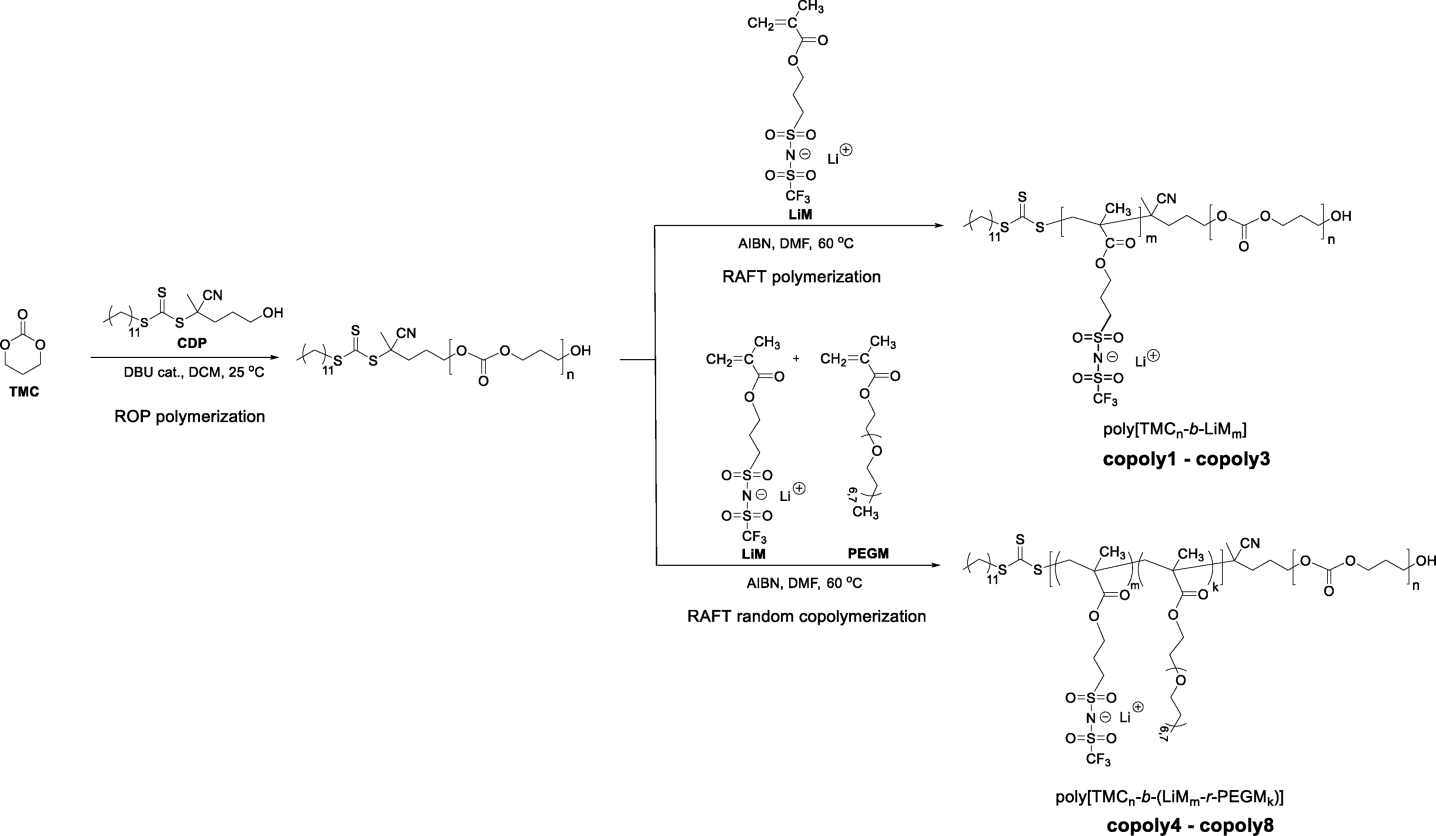
Synthetic Route for the Preparation of Poly[TMC*_n_*-*b*-LiM*_m_*] and Poly[TMC*_n_*-*b*-(LiM*_m_-r-*PEGM*_k_*)] Block Copolymers via the Subsequent Combination of ROP and RAFT Polymerizations

In this work, we focused on the development of novel poly[(ionic liquid)-*b*-(carbonate)] block copolymers (Scheme 1) with single Li-ion conducting features, showing greatly enhanced performance toward the state of the art of solid-state electrolyte systems, chiefly in terms of electrochemical stability and compatibility with both high voltage cathodes (NMC) and lithium metal anode, which resulted in reversible cycling near theoretical capacity in lab-scale Li-metal cells. Moreover, the addition of the polycarbonate block to the poly(ionic liquid) resulted in the significant enhancement of the mechanical properties of the resultant block copolymer electrolytes. For this purpose, the reported approach^45,46^ consisting of the utilization of the RAFT-agent having a terminal hydroxyl group as a dual initiator was applied, which allowed the subsequent realization of ring opening polymerization (ROP) of trimethylene carbonate (TMC) monomer and reversible addition-fragmentation chain transfer (RAFT) polymerization of methacrylic monomers. In the first step, ROP of TMC was exploited using 4-cyano-4-(dodecylsulfanylthiocarbonyl)sulfanylpentanol (CDP) as the initiator and 1,8-diazabicyclo(5.4.0)undec-7-ene (DBU) as the catalyst for the preparation of the poly(carbonate)-based macro-RAFT precursor (Scheme 1). It was further utilized in the second step for the synthesis of different series of block copolymers comprising lithium 1-[3-(methacryloyloxy)propylsulfonyl]-1-(trifluoromethylsulfonyl)imide (LiMTFSI) and poly(ethylene glycol) methyl ether methacrylate (PEGM) via RAFT polymerization. The resulting block copolymer electrolytes showed significantly improved mechanical properties (3 to 4 order of magnitude increase in storage moduli, at both 25 and 70 °C, compared to random copolymer based on LiMTFSI and PEGM), high oxidative stability (≥4.8 V vs Li^+^/Li at 70 °C), enhanced compatibility with the composite cathode components (NMC, LFP, electronic conductive additives) as well as the lithium metal anode, high Li-ion transport to enable high power, and excellent plating/stripping ability with resistance to dendrite growth.

## Results and Discussion

### Ring Opening Polymerization (ROP) of Trimethylene Carbonate (TMC)

Among the living polymerization techniques accessing polycarbonates with desired molar mass and well-defined end groups, ROP stands out as one of the leading approaches.^47^ Recent advances in the ROP of cyclic carbonates allowed elaborating a new metal-free green method, which uses various alcohols (benzyl alcohol, glycerol, propane-1,3-diol, etc.) as initiators and highly basic amines (1,8-diazabicyclo(5.4.0)undec-7-ene (DBU), 1,5,7-triazabicyclo[4.4.0]dec-5-ene (TBD), 4-dimethylaminopyridine (DMAP), etc.) as catalysts.^45,47,48^ This method was further expanded when RAFT chain transfer agents containing a hydroxyl functionality like 4-cyano-4-(dodecylsulfanylthiocarbonyl)sulfanylpentanol (CDP) or (*S*)-2-cyano-5-hydroxypentan-2-yl benzodithioate were exploited as dual initiators, allowing the subsequent realization of ROP and RAFT polymerizations.^45,46^ Such an approach was mainly applied to lactides^46,49^ and, to a lesser extent, to cyclic carbonates.^45,46^ Thus, the present work starts with a thorough investigation of TMC polymerization using CDP as the initiator and DBU as catalyst (Scheme 1 and Table S1 in the Supporting Information).

At first, the targeted degree of polymerization was fixed to 200, while the initiator-to-catalyst (CDP:DBU) molar ratio was varied from 0.05:1 to 1:1 (Table S1, ROP1 to ROP6). The poly(TMC) molar mass vs CDP:DBU molar ratio dependence was found to have a certain maximum on the curve at a low initiator-to-catalyst ratio (Figure S1). The maximum molar mass for poly(TMC) was found to be 16,400 g mol^–1^, which was observed at a 0.2:1 CDP:DBU molar ratio (Table S1, ROP3). Correspondingly, *M*_n_ determined experimentally even in the best run was lower than the targeted one (20,420 g mol^–1^). Except for 0.05:1, all other CDP:DBU ratios were capable of performing ROP of TMC with sufficiently low polydispersity index (*M*_w_/*M*_n_) varying from 1.17 to 1.40 (Table S1, ROP1–ROP 6). Further, different degrees of TMC polymerization were attempted at a fixed CDP:DBU ratio equal to 0.2:1 (Table S1, ROP7–ROP10 and ROP3). All the experiments were conducted at high monomer conversion (85–90%) and/or reaction completion. The experimental vs targeted *M*_n_ dependence was found to be close to the linear theoretical one only until the degree of polymerization reached 150, while for *M*_n_ above 15,000 g mol^–1^, the experimental values significantly deviated from the straight line, as further evidence of extreme character polymerization (Figure S2). The *M*_w_/*M*_n_ values gradually decreased with increasing molecular weight up to *M*_n_ = 20,000 g mol^–1^, which indicates an absence of molecular scrambling.^45^ It should be mentioned that, for all obtained poly(TMC) samples, the GPC-SEC chromatograms from a refractive index (RI) detector showed a small shoulder in the high-molar-mass region (Figure S3), likely accounting for the loss of polymerization control due to the high activity of the DBU catalyst.^45^

The influence of the solvent on ROP of TMC was evaluated by synthesizing poly(TMC) in dichloromethane (DCM), tetrahydrofuran (THF), and toluene (ROP 9, ROP11, and ROP12, respectively, in Table S1). While in DCM and THF the reaction occurred in solution, the nascent poly(TMC) started to precipitate in toluene after 5 h of reaction, thus reducing the isolated yield to 15% (Table S1, ROP12). Comparing ROP in THF and DCM, we concluded that the experimental mass of poly(TMC) obtained in DCM was closer to the targeted one (*M*_n DCM_ = 16,000, *M*_n THF_ = 10,200, *M*_n target_ = 15,315 g mol^–1^), while the yields of reaction were higher (50 and 82%, respectively).

To summarize, the following reaction parameters were found to be optimal to synthesize poly(TMC) with highest molecular weight and low polydispersity: 22 °C, 40 h, 0.2:1, and 0.25 g ml^–1^, in temperature, duration of reaction, CDP:DBU molar ratio, and TMC concentration, respectively, and DCM as a solvent (Table S1, ROP9).

Once the optimal conditions were established, the synthesis of poly(TMC) was scaled up and the macro-chain transfer agent was obtained with a molecular weight of 18,300 g mol^–1^ (GPC in THF) or 20,100 g mol^–1^ (GPC in 0.1 M LiTFSI in DMF). The structure of poly(TMC) was confirmed by H^1^and C^13^ NMR and IR spectra (Figures S4–S6). H^1^ NMR showed the desired end-groups and no indication of competitive side reactions. 4-Cyano-4-[(dodecylsulfanylthiocarbonyl)sulfanyl]pentane carbonate was clearly observed at the α-chain position, while the peaks related to the main chain were slightly shifted upfield relative to TMC monomer (Figure S4). Complete assignments for H^1^ NMR are the following: 4.23 ppm (TMC repeating unit: *CH_2_*CH_2_*CH_2_*), 2.04 ppm (TMC repeating unit: CH_2_*CH_2_*CH_2_), 3.7 ppm (terminal TMC unit: *CH_2_*OH), and 3.31, 1.92, 1.87, 1.64, 1.38, 1.25, 0.87 ppm (CDP moiety). The molar mass of poly(TMC) determined by NMR (*M*_n NMR_ = 16,999 g mol^–1^) was found to be in line with GPC observations. The FTIR spectrum of poly(TMC) presents the characteristic vibration bands of polycarbonate, as depicted in Figure S6. The peaks at 2975 and 2916 cm^–1^ were associated with aliphatic −CH– stretching. The strong bands at 1740 (C=O stretching), 1239 (asymmetric), and 1033 cm^–1^ (symmetric) C–O–C stretching were assigned to the carbonate linkage. Some bands from the CDP terminal unit are found at 1477 (asymmetrical −CH_3_ stretching), 1086 (stretching vibrations of C=S in (RS)_2_C=S), and 722 cm^–1^ (pendulum vibrations of −(CH_2_)*_n_*– at *n* > 4).

### RAFT Synthesis of Poly[TMC*_n_*-*b*-LiM*_m_*] and Poly[TMC*_n_*-*b*-(LiM*_m_*-*r*-PEGM*_k_*)] Block Copolymers

The investigation of block copolymer synthesis started with the RAFT polymerization of LiM monomer (LiMTFSI) using the poly(TMC) precursor as the macro-RAFT transfer agent and AIBN as the initiator. DMF was chosen as a solvent due to its ability to dissolve both poly(TMC) and LiM as well as because its utilization in (co)polymerization of LiM monomer allowed for achieving high yields and high molecular weights of the resulting polyelectrolytes.^20,35,50−52^ Using the optimal reaction conditions determined previously for RAFT polymerization of LiM^35^ ([AIBN]:[macro-RAFT] = 1:5 by mol, [DMF]:[poly(TMC) + LiM] = 3:1 by weight), a set of poly[TMC*_n_*-*b*-LiM*_m_*] block copolymers (Table 1, copoly1–3) targeting different degrees of polymerization, were successfully prepared. The obtained ionic block copolymers were firm and densely packed yellowish rubber-like materials. The GPC-SEC analysis of poly[TMC*_n_*-*b*-LiM*_m_*] block copolymers in 0.1 M LiTFSI solution in DMF revealed the increase of *M*_n_ values in comparison with the initial poly(TMC), while the *M*_w_/*M*_n_ ratios ranging between 1.32 and 1.36 were found to be satisfactory (Table 1, copoly1–3). The SEC traces in Figure S3 show the shifting of molecular weights to a higher region under preservation of the distribution. The GPC-SEC chromatograms of all investigated block polymers exhibit single symmetrical peaks (Figure S3). Despite a relatively good yield of copolymers (67–69%), the determined *M*_n_ values were less than the theoretical ones (Table 1, copoly1–3). This result correlates with the previously observed trend in the underestimation of molecular weights for LiM-based copolymers via GPC.^33,35^

**Table 1:** Selected Properties of the Poly(TMC) Macro-RAFT Agent and Poly[TMC*_n_*-*b*-LiM*_m_*] Copolymers Obtained by RAFT Polymerization

		poly[TMC*_n_*-*b*-LiM*_m_*] (A-*b*-B copolymer)								
	poly(TMC) (A-block)	B-block	A-*b*-B copolymer							
					σ (S cm^–1^)					
polymer	*M*_n_ (SEC)^a^ (g mol^–1^)	*M*_n_ (target)	*M*_n_ (SEC) (g mol^–1^)	*M*_w_/*M*_n_ (SEC)	25 °C	70 °C	[TMC]/[Li^+^]^b^	*T*_g1_ (°C)^c^	*T*_g2_ (°C)^c^	*T*_onset_ (°C)^d^
poly(TMC)	20,100							–15		175
copoly1		5000	21,100	1.31	9.5 × 10^–10^	1.3 × 10^–7^	66	–14		190
copoly2		9000	23,050	1.32	2.2 × 10^–11^	2.0 × 10^–8^	23	–14	140	190
copoly3		18,000	24,700	1.36	8.4 × 10^–11^	5.6 × 10^–8^	15	–14	140	205

a By GPC in 0.1 M solution of LiTFSI in DMF at 50 °C, *M*_w_/*M*_n_ = 1.29 (*M*_n_ = 18,400 g mol^–1^, *M*_w_/*M*_n_ = 1.19 by GPC in THF at 40 °C).

b Molar ratio calculated considering the experimentally determined molar masses.

c By DSC.

d By TGA in air.

One should note here that molecular weights were calculated via conventional calibration and are referenced to PMMA standards. Otherwise, the high electrostatic repulsion between monomer units in the formed polymer can slow down the homopolymerization of LiM. The long duration of the reaction (48 h) and polymer yields below 85–90% likely account for the abovementioned second explanation.

The structure and purity of poly[TMC*_n_*-*b*-LiM*_m_*] block copolymers were supported by ^1^H, ^13^C, and ^7^Li NMR and IR spectroscopies (Figures S7, S8, and S9). ^1^H NMR signals at 4.14 and 1.95 ppm were attributed to the poly(TMC) chain and a set of residual protons for the end-groups coming from the initial CDP RAFT agent (Figure S7). New signals at 4.01, 3.00, and 1.97 ppm assigned to COOCH_2_, CH_2_SO_2_, and CH_2_CH_2_SO_2_ of the poly(LiM) side chain as well as broad signals at 2.0–1.6 and 0.92 and 0.75 ppm that correspond to CH_2_ and CH_3_ groups of the poly(LiM) backbone were clearly observed (Figure S7a).

Except for signals attributed to poly(TMC) block and end-groups from the CDP RAFT agent, ^13^C NMR demonstrates signals assigned to poly(LiM) block, namely, at 176.84 and 176.27 (CO_2_ methacrylate), 120.15 (CF_3_), 63.30 (SO_2_-CH_2_-CH_2_-CH_2_O), 53.55 (br, CH_2_ methacrylate), 51.11 (CH_2_SO_2_), 44.21 (CCH_3_ methacrylate), 23.00 (CH_2_CH_2_SO_2_), and 17.94 and 16.56 ppm (CH_3_ methacrylate) (Figure S7b). Eventually, the CF_3_ group provides a singlet at −79.7 ppm in ^19^F NMR (Figure S8a), and the Li cation is observed as a singlet at 3.7 ppm in ^7^Li NMR (Figure S8b). The IR spectra of copolymers showed the absorption bands at 2973 and 2917 cm^–1^ assigned to CH_2_ stretching, 1743 cm^–1^ recognized as vibrations of the C=O group, and 1243 and 1033 cm^–1^ attributed to the asymmetric and symmetric C–O–C vibrations of the ester groups, respectively (Figure S9). The characteristic bands of the sulfonylimide anion were observed at ∼1330 (asymmetric S=O), ∼1190 (symmetric S=O), and ∼1062 (CF) cm^–1^ (Figure S9).

**Figure 1:**
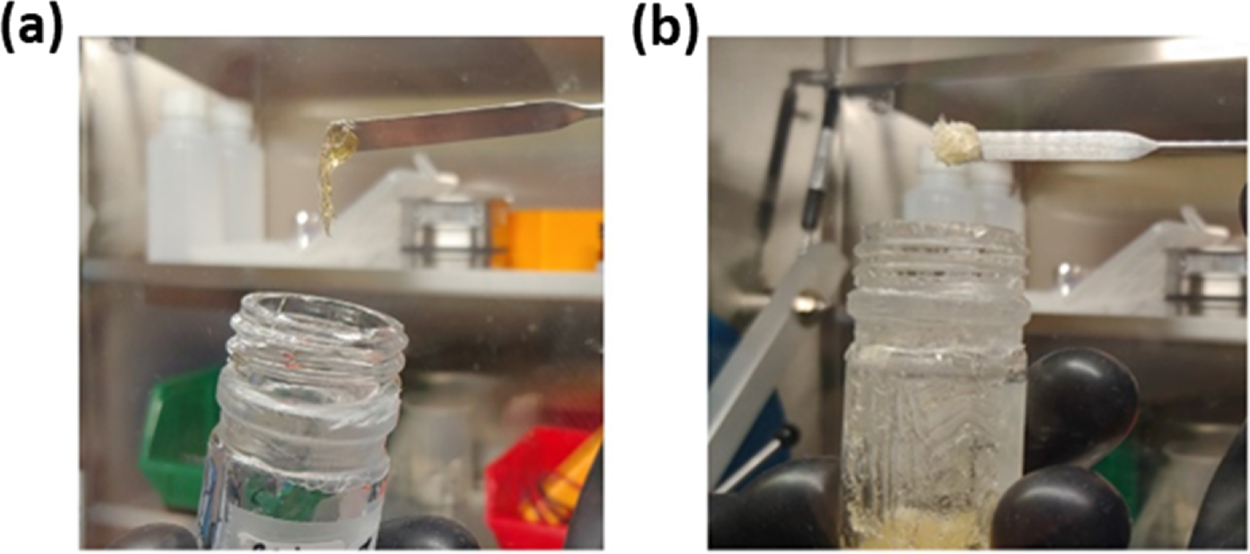
Digital photographs showing the appearance of poly[LiM*_m_*-*r*-PEGM*_k_*] (a) and poly[TMC*_n_*-*b*-(LiM*_m_*-*r*-PEGM*_k_*)] copoly8 (b) inside an argon-filled dry glove box.

We demonstrated previously^33,35,51^ that the highest ionic conductivity for linear methacrylate-based SICPs can be achieved by copolymerization of lithium ion containing monomers with PEGM. The presence of oxyethylene fragments in the side chain of poly(PEGM), by analogy to PEO,^53^ significantly improves the solubility of ionic species, thus facilitating their dissociation and correspondingly enhancing the ionic conductivity of the resulting copolymers. The study was further focused to the synthesis of poly[TMC*_n_*-*b*-(LiM*_m_*-*r*-PEGM*_k_*)] (Scheme 1) using the same conditions of poly[TMC*_n_*-*b*-LiM*_m_*] synthesis. Random RAFT copolymerization was used to prepare a set of block copolymers with the fixed length of poly(TMC) block and variable size of poly(LiM*_m_*-*r*-PEGM*_k_*) extension at different LiM:PEGM molar ratios (Table 2, copoly4–copoly8). The resulting poly[TMC*_n_*-*b*-(LiM*_m_*-*r*-PEGM*_k_*)] ionic block copolymers are yellow rubber-like materials (Figure 1b), which were a bit softer than poly[TMC*_n_*-*b*-LiM*_m_*] and harder than poly(LiM*_m_*-*r*-PEGM*_k_*) (Figure 1a). The GPC-SEC trace of poly[TMC*_n_*-*b*-(LiM*_m_*-*r*-PEGM*_k_*)] exhibits single symmetrical peaks and clearly demonstrates the shift of molecular weights to the higher region (Figure S3). As in the case of poly[TMC*_n_*-*b*-LiM*_m_*], the *M*_n_ values determined for poly[TMC*_n_*-*b*-(LiM*_m_*-*r*-PEGM*_k_*)] were lower than the theoretical ones (Table 2), while the *M*_w_/*M*_n_ ratios remained satisfactorily low in the range of 1.22–1.39, demonstrating the control over polymerization (Table 2, copoly4–copoly8). The LiM:PEGM molar ratios determined by NMR were higher than the loaded ones, showing higher reactivity of PEGM monomer (Table 2).

**Table 2:** Selected Properties of Poly[TMC*_n_*-*b*-(LiM*_m_*-*r*-PEGM*_k_*)] Copolymers Obtained by Random RAFT Copolymerization Also Compared with Different Polymeric Systems

			poly[TMC*_n_*-*b*-(LiM*_m_*-*r*-PEGM*_k_*)] (A-*b*-B copolymer)									
	poly(TMC) (A-block)		B-block		A-*b*-B copolymer							
								σ (S cm^–1^)				
polymer	*M*_n_ (SEC)^a^ (g mol^–1^)	*M*_w_/*M*_n_ (SEC)^a^	LiM/PEGM mol. ratio (target)	*M*_n_ (target)	LiM/PEGM mol. ratio (NMR)^b^	*M*_n_ (SEC)^c^ (g mol^–1^)	*M*_w_/*M*_n_ (SEC)^c^	25 °C	70 °C	*T*_g1_ (°C)^d^	*T*_g2_ (°C)^d^	*T*_onset_ (°C)^e^
copoly4	20,100^f^	1.29^f^	1:2	20,000	1:3.3	29,730	1.24	1.4 × 10^–7^	3.6 × 10^–6^	–35	–16	
copoly5			1:2	30,000	1:2.9	34,120	1.33	1.0 × 10^–7^	2.8 × 10^–6^	–36	–16	
copoly6			1:5	15,000	1:9.0	25,450	1.26	1.1 × 10^–7^	1.9 × 10^–6^	–50	–16	
copoly7			1:5	20,000	1:8.3	30,480	1.22	2.9 × 10^–7^	3.7 × 10^–6^	–49	–16	165
copoly8			1:5	30,000	1:8.1	34,600	1.39	1.1 × 10^–7^	2.9 × 10^–6^	–51	–16	155
poly(PEGM)^g^	23,600	1.16								–62		160
poly(LiM)^g^	52,700	1.20						1.1 × 10^–12^		105		250
poly(LiM*_m_*-*r*-PEGM*_k_*)	30,600	1.14	1:5		1:6.8			2.3 × 10^–7^		–31		170

a By GPC in 0.1 M LiTFSI in DMF at 50 °C.

b By NMR in DMSO-*d*_6_ at 25 °C.

c By GPC in 0.1 M LiTFSI in DMF at 50 °C.

d By DSC.

e By TGA in air.

f *M*_n_ = 18,400 g mol^–1^ and *M*_w_/*M*_n_ = 1.19 by GPC in THF at 40 °C.

g For comparison from ref (35).

The structure and purity of poly[TMC*_n_*-*b*-(LiM*_m_*-*r*-PEGM*_k_*)] were confirmed by ^1^H, ^13^C, ^19^F, and ^7^Li NMR, as well as IR spectroscopy and elemental analysis (Figures S9–S12). Apart from signals attributed to poly(TMC) block, end-groups from the CDP RAFT agent, and poly(LiM), the ^1^H NMR of poly[TMC*_n_*-*b*-(LiM*_m_*-*r*-PEGM*_k_*)] contains signals at 3.52 and 3.24 ppm, assigned to OCH_2_CH_2_O and CH_3_O fragments of PEGM, respectively (Figure S10). The determined featuring signals in ^13^C NMR are as follows: 71.28 (CH_2_OCH_3_), 69.79 (CH_2_OCH_2_ EG), 69.59 (CH_2_CH_2_OCH_3_), 67.82 (COOCH_2_CH_2_O), and 58.01 (CH_3_O) (Figure S11). ^19^F and ^7^Li NMR show single peaks at −79.7 and −0.98 ppm, respectively (Figure S12). The IR spectra of copolymers differ from poly[TMC*_n_*-*b*-LiM*_m_*] by the presence of absorption bands at 1119 and 862 cm^–1^ assigned to aliphatic ether −C–O– and −CH_2_–CO– stretching, respectively (Figure S9).

### Thermal Properties

The thermal stability of an electrolyte is fundamental in determining the safety of practical LiBs. Actually, the accidental overheating of the battery during the charge/discharge process may cause decomposition reactions of the electrolyte followed by an unwanted and uncontrollable temperature increase, known as “thermal runaway”, which eventually leads to hazardous battery failure.^54^

**Figure 2:**
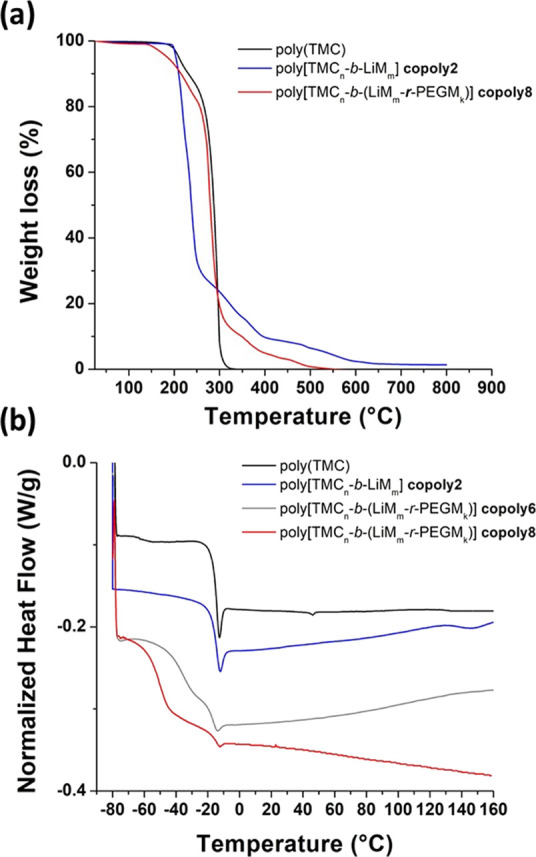
TGA (a) and DSC (b) traces of poly(TMC), copoly2, copoly6, and copoly8 (TGA was performed under air flow at a heating rate of 5 °C min^–1^).

The determination of the temperature operation limits for ionic block copolymers was assessed by thermogravimetric analysis. According to TGA (Figure 2a and Figure S13), the onset mass loss temperature (*T*_onset_) for poly(TMC) was found to be 175 °C, while it exceeded 250 °C for poly(LiM).^35^ The *T*_onset_ values of the poly[TMC*_n_*-*b*-LiM*_m_*] decrease accordingly to the following order with respect to the ionic part content (Table 1):
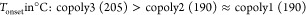


The thermal stability of poly[TMC*_n_*-*b*-(LiM*_m_*-*r*-PEGM*_k_*)] block copolymers was mainly governed by the degradation of TMC and PEGM parts, being those having the lowest thermal stability limit (*T*_onset_ = 175 and 160 °C, respectively). As a result, all poly[TMC*_n_*-*b*-(LiM*_m_*-*r*-PEGM*_k_*)] block copolymers independent of their composition possessed similar onset loss temperatures in the range of 155–165 °C (Figure 2a and Table 2). Overall, these values are particularly attractive for application in practical Li-based batteries and account for remarkably safer characteristics than conventional liquid electrolyte based devices, which become thermally unstable already above 80 °C.^55^

The glass transition temperatures of copolymers were determined by DSC (Figure 2b, Tables 1 and 2). The starting block poly(TMC) macro-RAFT agent with *M*_n_ = 20,100 g mol^–1^ showed a *T*_g_ of −15 °C (Figure 2b). The copoly1, having in accordance with GPC the PC:LiM unit ratio equal to 197:3, is characterized by only one *T*_g_ at −14 °C. The growth of the LiM block in copoly2 and copoly3 resulted in the appearance of the second *T*_g2_ at around 140 °C, which corresponds to the transition temperature of neat poly(LiM) observed at 105 °C (Table 1 and Figure 2b).^35^

The RAFT random copolymerization of PEGM and LiM in different ratios led to the significant decrease in *T*_g_ of the obtained poly[TMC*_n_*-*b*-(LiM*_m_*-*r*-PEGM*_k_*)] block copolymers (Figure 2b and Table 2). It is worth noticing the presence of the two distinct glass transition temperatures for the copoly4–copoly8 samples (Table 2). The *T*_g2_, related to the poly(TMC) block, was constantly observed at −16 °C, while the *T*_g1_ values ranged from −36 to −51 °C in the following order:
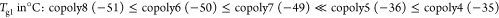


As for the above detailed data and considering the low glass transition temperature of the neat poly(PEGM) (−62 °C),^35^ we assume that the *T*_g1_ of poly[TMC*_n_*-*b*-(LiM*_m_*-*r*-PEGM*_k_*)] block copolymers is governed mainly by the PEGM:LiM molar ratio and is practically independent of the molar mass of copolymer (Table 2).

### Ionic Conductivity

Ionic conductivity (σ) values of polyelectrolytes as a function of the temperature were recorded by electrochemical impedance spectroscopy (EIS, Tables 1 and 2). Prior to EIS measurements, samples were heated at 60 °C (1 h) and, subsequently, equilibrated at 20 °C for 4 h to ensure optimal interfacial contact with the electrodes. First, ionic conductivities were determined for poly[TMC*_n_*-*b*-LiM*_m_*] block copolymers (Table 1). At 25 °C, σ values increased from 8.4 × 10^–11^ to 9.5 × 10^–10^ S cm^–1^ depending on the size of LiM block, arranged as follows in descending order:
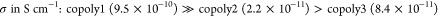


Thus, the higher both the *M*_n_ of the block copolymer and the size of the LiM block, the lower was the ionic conductivity of the polyelectrolyte. At 70 °C, σ values largely increased up to 2 × 10^–7^ S cm^–1^ while maintaining the same trend as above (Table 1). These are rather low values of ionic conductivity, likely ascribed to the limited chain mobility of the ionic block having high glass transition temperatures (*T*_g1_ = −16 °C, *T*_g2_ = 140 °C).

**Figure 3:**
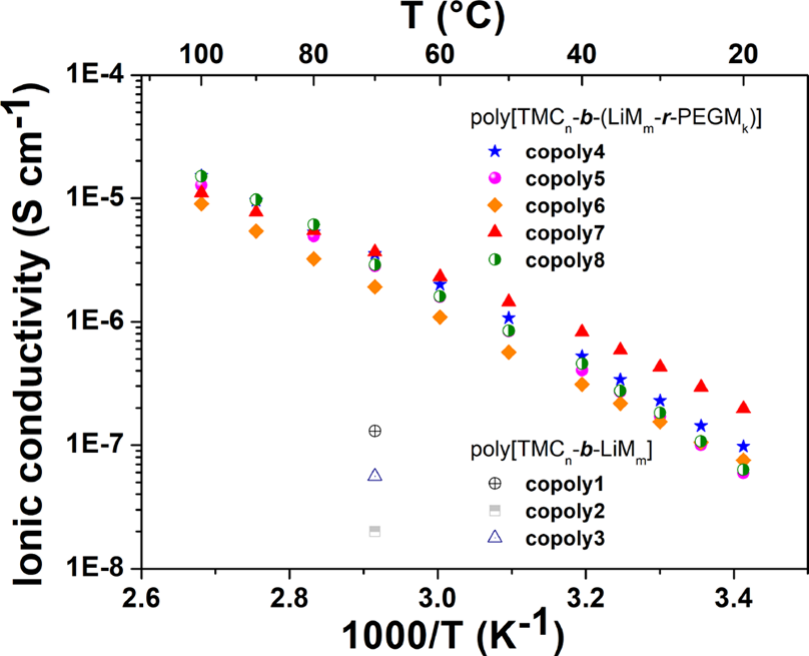
Arrhenius plot of ionic conductivity versus inverse temperature determined by EIS in the range of 20–100 °C for ionic block copolymers.

Representative plots of ionic conductivity vs temperature for poly[TMC*_n_*-*b*-(LiM*_m_*-*r*-PEGM*_k_*)] block copolymers are shown in Figure 3. The random copolymerization of LiM with PEGM during the growth of the second block allowed for significant ionic conductivity enhancement compared to the poly[TMC*_n_*-*b*-LiM*_m_*] polyelectrolyte samples. Indeed, all poly[TMC*_n_*-*b*-(LiM*_m_*-*r*-PEGM*_k_*)] block copolymers provided σ values exceeding 10^–7^ S cm^–1^ already at 25 °C. Ionic conductivity values were found to be similar in the range of 1.0 to 2.9 × 10^–7^ S cm^–1^, arranged as follows, depending on the composition of the (LiM-*r*-PEGM) second block:
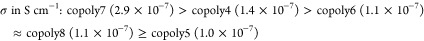


For all copolymers, ionic conductivity increased with increasing temperature up to about 10^–6^ S cm^–1^ already at 60 °C. Slight deviations from the linear Arrhenius behavior were observed (Figure 3), particularly for copoly7, indicating that lithium ion diffusion not only occurs through isolated hopping on the pendant sulfonamide groups but also results from local segmental motion of the coordination sites in the polymer main chain.

At 70 °C, the ionic conductivity values of the poly[TMC*_n_*-*b*-(LiM*_m_*-*r*-PEGM*_k_*)] block copolymers were found to be close, in the 1.9 to 3.7 × 10^–6^ S cm^–1^ range (Table 2). Thus, the choice of the optimal copolymer composition for further electrochemical tests was made on the basis of mechanical properties. In this respect, as for its efficient film forming ability, copoly8 was selected as the representative sample for scale up and further studies.

### Morphology

**Figure 4:**
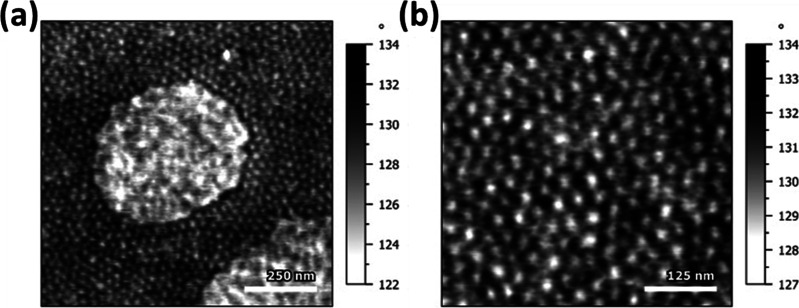
AFM images of the poly[TMC*_n_*-*b*-(LiM*_m_*-*r*-PEGM*_k_*)] copoly8 film at different resolutions (a, b).

Ionic (multi)block copolymers are known to show spontaneous formation of ordered micro- and nanosized structures, which actually contribute to the enhancement of both their ionic conductivities and mechanical properties.^43,56−60^ In poly[TMC*_n_*-*b*-(LiM*_m_*-*r*-PEGM*_k_*)] block copolymers (Table 2, copoly4–copoly8), two distinct glass transition temperatures were observed (Figure 2a); thus, the morphology of the representative copoly8 was investigated by atomic force microscopy (AFM). The AFM images of the phase shift revealed a native nanophase separation at the surface of the drop-cast film (Figure 4). A quasi-hexagonally packed cylinder arrangement perpendicular to the surface can be clearly observed. The phase shift can be here qualitatively linked to the stiffness of the surface, where the higher surface stiffness creates higher repulsive contact force, which, in turn, increases the resonance frequency/diminishes the phase shift. A nanophase attribution can be made on the cylinders, having 22.5 ± 2.5 nm diameter and representing the poly(TMC) phase, that are regularly distributed inside the matrix of poly(LiM*_m_*-*r*-PEGM*_k_*) with a 35.7 ± 4.5 nm pitch. The observed strong nanophase separation in copoly8 can be the hypothesized reason for maintaining the same ionic conductivity level as demonstrated by random poly(LiM*_m_*-*r*-PEGM*_k_*) despite having a less amount of free ion conducting species (Table 2).

### Rheology

**Figure 5:**
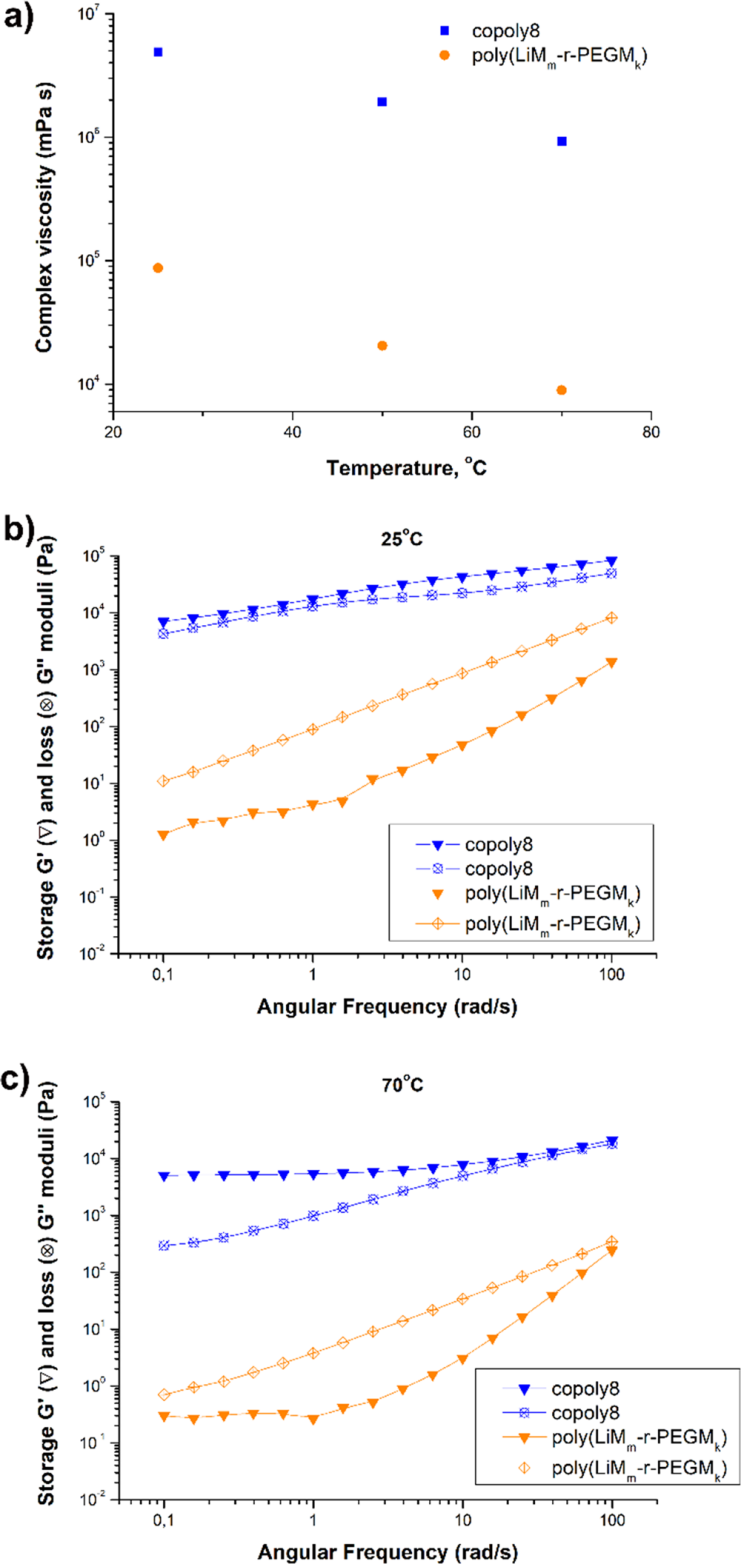
Temperature dependence of the complex viscosity (a) and frequency dependence of the storage modulus *G*′ (full symbols) and the loss modulus *G*″ (open symbols) obtained at 25 (b) and 70 °C (c) for poly[TMC*_n_*-*b*-(LiM*_m_*-*r*-PEGM*_k_*)] copoly8 and poly(LiM*_m_*-*r*-PEGM*_k_*).

The viscoelastic properties of the newly synthesized poly[TMC*_n_*-*b*-(LiM*_m_*-*r*-PEGM*_k_*)] (namely, copoly8) and previously reported poly(LiM*_m_*-*r*-PEGM*_k_*)^35^ were compared by carrying out rheological measurements in a small-amplitude oscillatory flow mode. The temperature dependence of complex viscosity at a constant frequency of 1 Hz is shown in Figure 5a. Both copolymers demonstrated a neat decrease in their complex viscosity with an increase in temperature from 25 to 70 °C.

However, copoly8 exhibited a much higher complex viscosity than poly(LiM*_m_*-*r*-PEGM*_k_*) in the whole temperature range of testing. The storage (*G*′) and loss (*G*″) modulus frequency dependence was also investigated (Figure 5b,c).

While poly(LiM*_m_*-*r*-PEGM*_k_*) showed higher liquid-like character (*G*″ > *G*′), the poly[TMC*_n_*-*b*-(LiM*_m_*-*r*-PEGM*_k_*)] demonstrated enhanced solid-like character (*G*′ > *G*″) at both 25 and 70 °C. In addition, the absolute values of *G*′ in the case of copoly8 were several orders of magnitude higher than those of poly(LiM*_m_*-*r*-PEGM*_k_*) under the same measurement conditions. These outcomes are definitely relevant considering that the increase of the solid polymer electrolyte modulus is reported to effectively suppress/limit the formation and growth of lithium dendrites.^5,61^ As the compared copolymers are both linear and of similar molecular weight (Table 2), the observed change in viscoelastic properties can only be attributed to the presence of the poly(TMC) block in copoly8. Being stiffer, the poly(TMC) block improves significantly the viscoelastic properties of poly[TMC*_n_*-*b*-(LiM*_m_*-*r*-PEGM*_k_*)] copolymer in comparison with poly(LiM*_m_*-*r*-PEGM*_k_*).

### Electrochemical Stability

**Figure 6:**
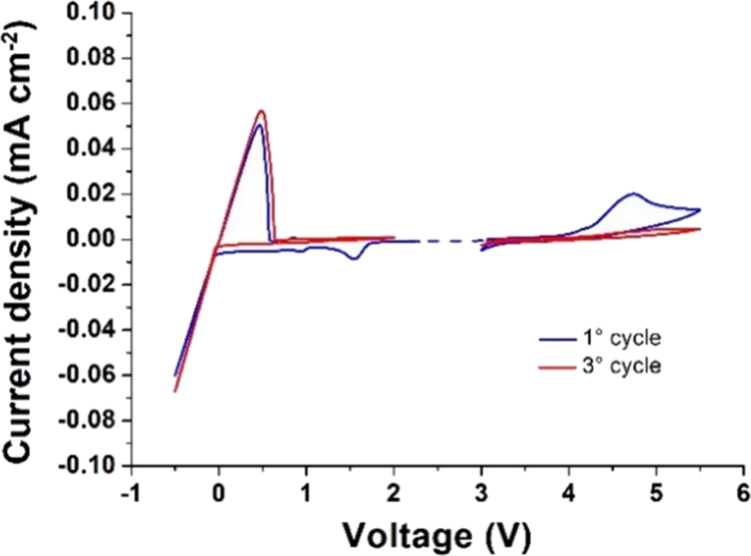
Electrochemical stability window for poly[TMC*_n_*-*b*-(LiM*_m_*-*r*-PEGM*_k_*)] copoly8 obtained by CV at 70 °C (stainless steel as the working electrode and Li foil as counter and reference electrodes, scan rate 0.1 mV s^–1^).

The electrochemical stability window (ESW) of the representative copoly8 sample was investigated by separate cathodic/anodic cyclic voltammetric (CV) scans at 70 °C (Figure 6). The slow scan rate (0.1 mV s^–1^) allowed for the detection of the faint reduction process, which was correlated with a small current flow just above 1 V vs Li^+^/Li, whereas the peak at about 1.5 V was likely ascribed to the decomposition of some electrolyte components, thus forming a passivating layer toward the lithium metal electrode, as well as to the reduction of some traces of side products from the synthesis. Well-defined and highly reversible lithium plating/stripping processes are clearly observable, as for the highly reversible couple of reduction/oxidation peaks between −0.5 and 0.5 V vs Li^+^/Li, which confirms the efficient transfer of lithium ions through the polymer network and at the polymer electrolyte/electrode interface. In the following anodic scan toward higher potential values, the possible oxidation of the electrolyte was ruled out, as for the absence of any detrimental oxidative processes below 4.2 V vs Li^+^/Li. The oxidation peak starting at above 4.2 V and closing at about 5 V vs Li^+^/Li in the first anodic scan was likely associated with the partial decomposition of ethylene oxide containing moieties in the polymer electrolyte. During the successive cycles, the intensity of the peaks largely decreased, which makes it difficult to identify any appreciable oxidative currents up to 5.5 V vs Li^+^/Li. In general, the anodic decomposition of an electrolyte is mainly connected to the oxidation of anions,^19^ but in the single-ion conducting polymer electrolyte under study, anions are chemically bonded to the polymer network. This assumption is supported by the presence of strongly anchored perfluorinated sulfonimide anions, covalently bonded to the polymer network that can be oxidized only at the electrolyte/electrode interface, hence accounting for the wide ESW (up to 5.5 V vs Li^+^/Li at 70 °C). This represents an excellent result for a single-ion polymer electrolyte, particularly at higher temperature that induces reactions that may even more severely affect the conditions of electrolyte stability,^62^ placing copoly8 on top of polyelectrolytes with highest electrochemical stability^16,63^ and supporting its safe practical use with high voltage cathodes.

### Lithium-Ion Transference Number and Compatibility with the Lithium Metal Electrode

The promising prospects of the newly developed single-ion conducting polymer electrolyte were further corroborated by testing copoly8 for its lithium-ion transference number (*t*_Li_^+^), determined by the methods of Evans et al.^64^ and Abraham et al.^65^ The resulting values from EIS and polarization experiments are given in Table S2. The typical Nyquist plot of a.c. impedance of a Li/copoly8/Li symmetrical cell at 70 °C is shown in Figure S14. The cell impedance did not change significantly during the experiment, and the limited initial resistance value of 670 Ω only decreased to 509 Ω, thus proving that a stable interfacial layer was readily formed at the interface with the lithium metal electrode.

The plot of the current response to the applied bias as a function of time is shown in Figure S15. A drop of less than 1 order of magnitude (from 7.66 to 7.08 μA) was observed before the steady state was reached. It resulted in a calculated *t*_Li_^+^ value of 0.91 (or 0.90 considering the changes in the bulk resistance and applying the modified version of the original Evans equation). It is worth noticing that both *t*_Li_^+^ values are noticeably close to unity and clearly significantly higher than standard liquid electrolyte containing salts, or RTIL-based electrolytes, or cationic PILs/Li salts and/or salt in polymers (e.g., PEO/Li), or composite electrolytes reported previously.^66,67^ The main factor that can be attributed to the deviation of the transference number value from unity is the nonzero mobility of anchored anions, chiefly due to the presence of a flexible, long spacer between anchored anions and the main chain and the overall inherent motion of polyanionic block since the test was conducted at temperature far above *T*_g_. Overall, t_Li_^+^ values for copoly8 are high enough to allow homogeneous lithium plating and stripping, thus preventing the formation and growth of inhomogeneous lithium dendritic structures and correspondingly guaranteeing safe and stable long-term operation, chiefly in lithium metal batteries.^5,68^

**Figure 7:**
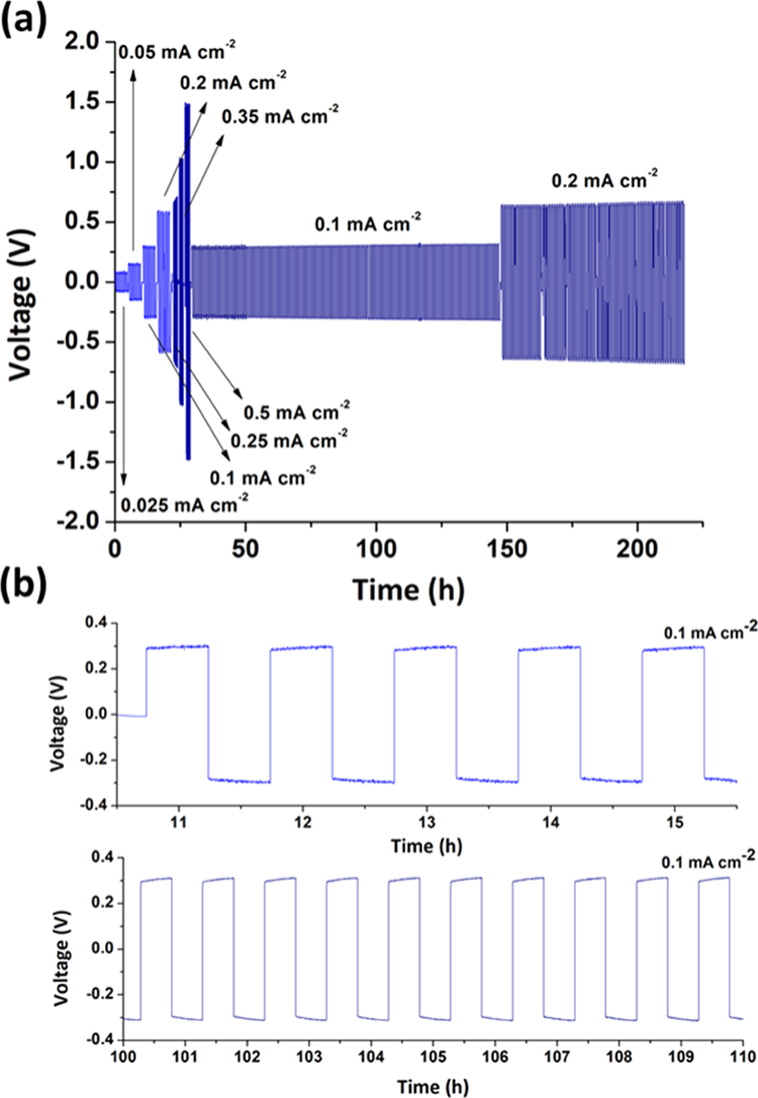
Voltage (V) vs test time (h) plots of Li stripping/plating for a symmetrical Li/copoly8/Li cell at various current densities (i.e., 0.025, 0.05, 0.1, 0.2, 0.25, 0.35, 0.5 mA cm^–2^) at 70 °C (a, b).

The stability/compatibility at the interface with the lithium metal electrode was confirmed by constant current (galvanostatic) reversible plating/stripping tests performed at 70 °C and increasingly higher current density values ranging from 0.025 to 0.5 mA cm^–2^ (30 min per step, see Figure 7). The novel single-ion conducting polymer electrolyte under study demonstrated excellent continuous reversible cycling for the whole accelerated test, without any detectable short circuit issues, even at relatively high 0.5 mA cm^–2^. Aiming at supporting the durability and safe operation of the SICP polyelectrolyte in lithium metal cells, a prolonged plating/stripping test was performed at a fixed current density regime of 0.1 mA cm^–2^ for 100 h followed by additional 100 h doubling the current to 0.2 mA cm^–2^ without observing either a large overpotential increase over time with respect to the initial value or any abrupt or unexpected current spikes/drifts, which can be related to irregular dendrite growth.

A deep understanding of the interfacial properties between the lithium metal electrode and the polymer electrolyte is fundamental for demonstrating the feasibility of SICP to operate steadily with a lithium metal electrode. For this purpose, a symmetrical Li/copoly8/Li cell was assembled and the electrolyte/Li metal electrode interfacial resistance was monitored over time at 70 °C. As shown in Figure S16, the copoly8-based lithium symmetrical cell showed a decrease of the bulk resistance in the first days of storage, mainly due to the temperature equilibration of the system. Remarkable stable interfacial resistance was obtained after few days, revealing the effective interfacial compatibility between the lithium metal electrode and the single-ion conducting polymer electrolyte under study.

### Electrochemical Behavior in the Li Metal Cell

**Figure 8:**
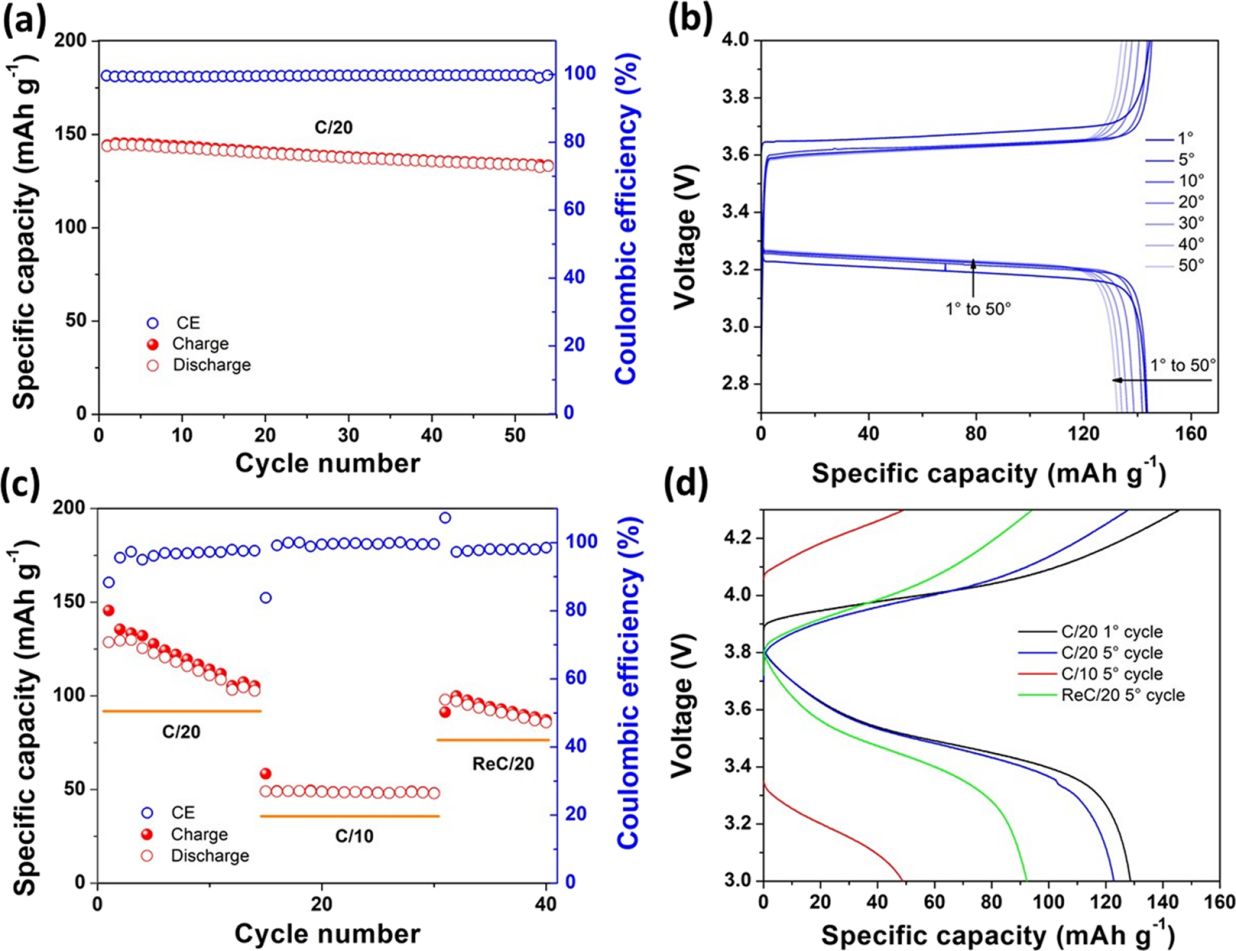
Galvanostatic cycling behavior of Li/copoly8/LFP (a, b) and Li/copoly8/NMC (c, d) solid-state cells at 70 °C. Specific capacity vs cycle number dependence (a) and corresponding charge/discharge voltage vs specific capacity profiles at a constant C/20 rate (b) of the Li/copoly8/LFP cell. Specific capacity vs cycle number dependence at C/20 and C/10 rates (c) and corresponding charge/discharge voltage vs specific capacity profiles at C/20 and C/10 rates (d) of the Li/copoly8/NMC cell.

Motivated by the promising results in terms of ion mobility, electrochemical stability, and interfacial compatibility, the newly prepared copoly8 polyelectrolyte was first assembled in a lab-scale lithium test cell with a lithium metal negative electrode (anode) and commercial LiFePO_4_ (LFP) as a reference active material for the positive electrode (cathode) in a Li/copoly8/LFP configuration. As detailed in the Supporting Information, the LFP-based electrode with a reasonably high active material loading of 4.17 mg cm^–2^ was obtained in the form of a catholyte using the same copoly8 as the active binder. The aim is to provide an ionically conducting interface between the SICP and the active material particles to enhance their “wettability” in the bulk of the electrode and to decrease the ion diffusion resistance at the electrode/electrolyte interface.^35^ Actually, unlike conventional batteries with liquid electrolytes, where the ion conduction inside the cathode is assured by homogeneous wetting of the electrolyte through the porosity of the composite electrode, in solid-state cells, the ionic conduction is often limited to the contact area at the interface with the SPE. To ensure proper drying, the obtained catholyte films were treated under high vacuum at 60 °C and stored for 48 h in a dry glove box prior to cell assembly. The cell was assembled using the neat copoly8 as the electrolyte without any further treatment of the electrodes or any plasticizers/enhancers (e.g., solvents, salts). The electrochemical behavior of the solid-state lab-scale cell (70 °C and C/20 rate, based on the theoretical specific capacity of the LFP active material) is shown in Figure 8a. It delivered stable and efficient charge/discharge cycling (>145 mAh g^–1^) at the first cycle, which corresponds to >91% of the practical specific capacity output (158 mAh g^–1^ at C/20) provided by the commercial LFP used as the active material when cycled with a standard LP30 liquid electrolyte. Excellent cycling stability and capacity retention were demonstrated upon prolonged cycling, with a very limited (≤2%) specific capacity drop after 10 cycles, and outstandingly high Coulombic efficiency (CE) approaching 100% during the whole cycling test. This is actually a remarkable result, particularly considering the active material loading, which is definitely high for a lab-scale polymer electrolyte cell^69^ and not too far from standard commercial cells.^70^ The excellent CE confirms the reversibility of the Li^+^ ion intercalation process and the stability of the obtained single-ion block polyelectrolyte. Very interestingly, no loss and even no gradual decrease of specific capacity during initial cycling were observed, which also accounts for the purity of the sample, its stability toward oxidation/reduction, compatibility with both electrode materials, and the formation of a stable passivation layer at the electrode/electrolyte interface. The remarkable electrochemical performance in terms of high capacity output and capacity retention after more than 50 consecutive charge/discharge cycles at a C/20 rate is likely ascribed to the efficient ion conduction in the polymer electrolyte separator and the favorable charge transport at the electrode/electrolyte interface in the cell. Figure 8b shows highly reversible and stable constant current potential versus specific capacity profiles, which nicely resemble the typical flat plateaus of the cathode corresponding to the Li^+^ ion deinsertion (charge) and insertion (discharge) from/in LiFePO_4_/FePO_4_.^71^ Clean and flat profiles with a sharp voltage drop at the end of the redox reaction related to the Li^+^ deinsertion/insertion mechanism suggest that polarization behavior during ion insertion/diffusion at the cathode/SICP interface was actually limited. The voltage difference between the charge and discharge potential plateaus was found to be in the order of 0.4 V, which is not negligible. The main cause of the voltage drop (overpotential) was assigned to the relatively low ionic conductivity and the intrinsic high bulk resistance of the polyelectrolyte. It is worth noticing here that the thickness of the electrolyte used in this proof-of-concept cell (∼100 μm) might have negatively affected the ion diffusion between the cathode and anode throughout the electrolyte; moreover, the commercial LFP used in this work is optimized to deliver high energy density rather than high power output.

We can assume that increasing the conductive carbon loading in the electrode may mitigate the overpotential issue; however, the optimization of the electrode formulation was beyond the scope of this work, which was indeed to show a proof-of-concept. Nonetheless, the voltage drop decreased while cycling (<0.3 V after 5 cycles), which accounts for a sort of activation of materials and amelioration of the interface while cycling due to the enhanced characteristics of the electrolyte.

With the purpose of confirming the excellent electrochemical stability at anodic potential values exceeding 4.8 V vs Li^+^/Li, copoly8 was further assembled in a lab-scale lithium cell prototype with a lithium metal negative electrode (anode) and a commercial LiNiMnCoO_2_ (NMC) as the active material at the positive electrode in a Li/copoly8/NMC configuration. The NMC-based cathode at a relatively high loading of 4.32 mg cm^–2^ was again obtained in the form of a catholyte using copoly8 as the active binder as detailed in the Supporting Information. The electrochemical behavior of the solid-state lab-scale cell was first studied in the voltage range between 3 and 4.3 V vs Li^+^/Li (70 °C, C/20 and C/10 constant current rates, based on the theoretical specific capacity of the NMC active material). The solid-state NMC-based cell delivered initial specific charge capacity values of 145 and 124 mAh g^–1^ after 1 and 6 cycles, respectively, at a C/20 rate (Figure 8c). Thus, no drastic capacity fade during initial cycling was observed, with a CE improving cycle-by-cycle (exceeding 95% after 5 cycles). Doubling the current rate to C/10, the specific discharge capacity delivered by the cell was still close to 50 mAh g^–1^ with only a slight overpotential increase compared to the potential vs specific capacity profile obtained at a lower rate (Figure 8d). This behavior was ascribed to the clear limitations associated with the internal resistance of the cell, as already observed for the LFP-based cell, mainly affected by the relatively high intrinsic resistance of the polyelectrolyte and the not engineered interface between the binder and the active material.

Again, we stress here that optimization in this respect was beyond the scope of this paper. Nonetheless, the proof-of-concept high-voltage cell demonstrated very good cycling stability, as confirmed by the almost complete specific capacity recovery while reducing the current rate to C/20 after 30 cycles and enhanced CE exceeding 97%. Yet, it is important to remark the performance of this proof-of-concept cell, being to our knowledge the first example of neat polycarbonate-based SICP operating in a truly solid-state Li-metal configuration with a high-energy 4 V-class NMC electrode without any performance enhancers, additives like plasticizers, and/or surface electrode treatment.

To support even more the high voltage stability of newly developed copoly8, a new Li/copoly8/NMC cell was assembled, which was stressed up to 4.8 V vs Li^+^/Li (70 °C, C/20 rate), while stepwise increasing the anodic voltage limit from 4.3 to 4.8 V by 0.1 V every 2 reversible constant current charge/discharge cycles. Representative potential vs specific capacity profiles extracted from the galvanostatic cycling test are shown in Figure S17 in the Supporting Information. A clear charge profile typical of lithium ion extraction was detected up to 4.8 V vs Li^+^/Li, thus confirming the very high voltage stability and cycling performance of the newly developed SICP.

**Figure 9:**
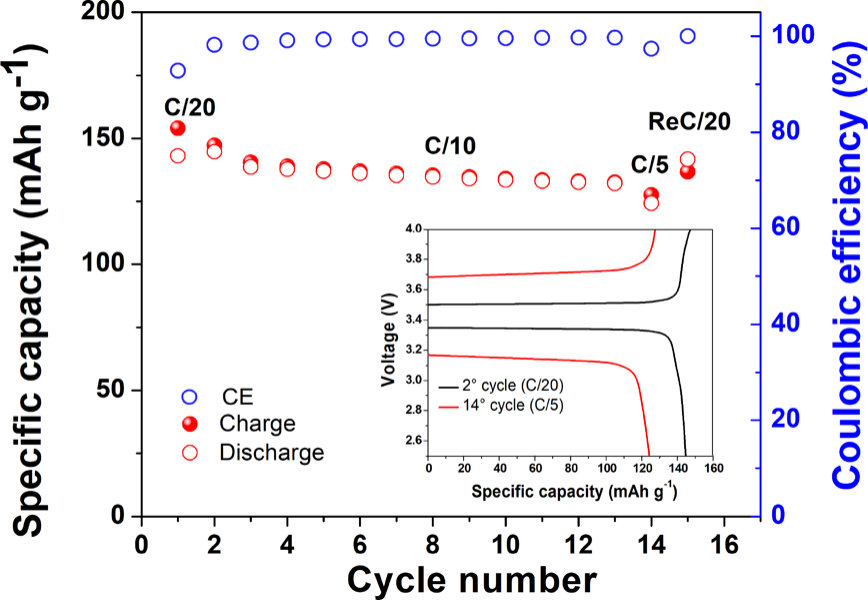
Galvanostatic cycling behavior at C/20, C/10, and C/5 rates upon charge and discharge at 70 °C of the Li/copoly8-PC/LFP lab-scale cell: specific capacity vs cycle number plot, including Coulombic efficiency values. The inset shows the charge/discharge potential profiles vs specific capacity at different constant current rates.

As already demonstrated in a previous work,^19^ the cross-linking technique allows the synthesis of SPE with embedded plasticizer components such as solvents and/or oligomers, enhancing cycling performance without detrimentally compromising the thermal stability of the polymer electrolyte and, thus, the safety of the final device.^19^ Following the same route, but without drastically altering the identity of this work, an additional Li/copoly8-PC/LFP lab-scale cell was assembled (the amount of PC was fixed at below 8 wt % with respect to the total mass of the polyelectrolyte in the cell). The results of the constant-current cycling test are shown in Figure 9. It is well evident that the new cell showed an outstandingly reduced capacity drop compared to the previous LFP-based truly solid-state cell while doubling the current regime to C/10 and even up to C/5 without any remarkable drop of specific capacity. Moreover, the voltage profiles remained flat and stable with very limited overpotential at the C/20 rate, which accounts for a greatly reduced electrode/electrolyte resistance, as shown in the inset of Figure 9. The overpotential increased while cycling at C/5, but still, the profile remained flat with no sign of enhanced sloping of the curve and very limited polarization, thus accounting for the largely enhanced ion conduction in the polymer electrolyte separator and the more favorable charge transport between the electrodes and the electrolyte in the new cell even with a very limited amount of added plasticizer.

The CE exceeded 99% during initial and prolonged cycling at low as well as at high rates, thus confirming the reversibility of the lithium ion intercalation process and the electrochemical and interfacial stabilities of the single-ion conducting block copolymer electrolyte.

## Conclusions

In this work, we reported the synthesis of novel solid-state polyelectrolytes based on poly(carbonate)-*b*-poly(ionic liquid) with single Li^+^ ion conducting features. The single-ion conducting polyelectrolytes were purposely modified by designing novel block copolymers that combine one block responsible for high ionic conductivity and the second block for improved mechanical properties and outstanding electrochemical stability. Such ionic block copolymers were obtained by subsequent ring opening polymerization (ROP) and reversible addition-fragment chain-transfer (RAFT) polymerization techniques. At first, trimethylene carbonate monomer was polymerized by ROP, using a RAFT-agent having a hydroxyl terminal group, and in the second step, the prepared poly(carbonate)-based macro-RAFT precursor was used for random RAFT (co)polymerization of lithium 1-[3-(methacryloyloxy)propylsulfonyl]-1-(trifluoromethylsulfonyl)imide (LiM) and poly(ethylene glycol) methyl ether methacrylate. Materials were thoroughly characterized from the physicochemical viewpoint with a complete bunch of techniques.

The new generation of SICPs, namely, poly[TMC*_n_*-*b*-(LiM*_m_*-*r*-PEGM*_k_*)] block copolymers, differs from the previously reported single-ion conducting block copolymer electrolytes reported by our group by the presence of poly(TMC) block. Remarkably, while maintaining sufficient ionic conductivity and high lithium-ion transference number (0.91), the poly(TMC) block imparted outstanding electrochemical stability up to ∼5 V vs Li^+^/Li at 70 °C and significantly enhanced the viscoelastic properties of poly[TMC*_n_*-*b*-(LiM*_m_*-*r*-PEGM*_k_*)] block copolymers. The novel block copolymers demonstrated ionic conductivities up to 3 × 10^–7^ and 4 × 10^–6^ S cm^–1^ at 25 and 70 °C, respectively. Such a reasonably high level of ionic conductivity for an SICP was explained by several factors, including (i) the presence of oxyethylene fragments, which significantly improve the solubility of ionic species and facilitate their dissociation, (ii) the two low *T*_g_ enabling motion of polymer chains, and (iii) the nanophase separation of the cast films responsible for mechanical integrity and effective prevention of dendrite growth.

The proof-of-concept lab-scale truly solid-state Li-metal cells assembled with such novel ionic block copolymers using both standard LFP and high voltage NMC-based composite electrodes at relatively high active material loading provided excellent performances in terms of high specific capacity output, stability, and reversible cycling even up to 4.8 V vs Li^+^/Li. This is one of the most relevant results so far among the literature reports on truly solid-state single-ion conducting systems, which postulates the implementation of this family of polyelectrolytes in next-generation advanced, all-solid Li-metal batteries, conceived for high energy and/or power applications at enhanced safety.

Further improvements should include the enhancement of polyelectrolyte neat ionic conductivity and the engineering of effective electrode/electrolyte interfaces, along with the realization of self-standing films for ease of designing, processing, and device scale-up. However, the comprehensive study carried out in this work on the novel materials and innovative approach, including the nonconventional use of the hydroxyl-terminated RAFT agent, opens up new promising paths on the dynamics of dual ROP-RAFT polymerization and will largely support the scientific community on the development of safe, electrochemically stable polycarbonate-based solid-state electrolytes allowing advanced Li-metal based battery technologies to intrude the HEV/EV market in the next decade.

## Supporting Information Available

Full experimental procedures, characterization data including ^1^H, ^13^C, ^19^F, and ^7^Li NMR and IR spectroscopy, pseudo-kinetic plots for ROP of TMC, GPC-SEC chromatograms, TGA curves, Li ion transference number analysis, interfacial stability analysis of copoly8 sandwiched between two lithium metal electrodes, charge/discharge potential vs specific capacity profiles for Li/copoly8/NMC cell at high voltage, and supplementary tables (PDF)
