# Supplementary material for: Unique Carbonate-Based Single Ion Conducting Block Copolymers Enabling High-Voltage, All-Solid-State Lithium Metal Batteries
Source: Macromolecules. 2021 Jul 14;54(14):6911–24. doi: 10.1021/acs.macromol.1c00981 (PMC8397401; doi:10.1021/acs.macromol.1c00981)
Supplement: Supplementary file 1 — ma1c00981_si_001.pdf [file ma1c00981_si_001.pdf]

Supporting Information

Unique carbonate-based single ion conducting block copolymers enabling high-voltage, all-solid-state lithium metal batteries

Gabriele Lingua[a,b], *Patrick Grysan*[c], *Petr S. Vlasov*[d], *Pierre Verge*[c], *Alexander S. Shaplov*[c]*, *Claudio Gerbaldi* [a,b]*

[a] GAME Lab, Department of Applied Science and Technology (DISAT), Politecnico di Torino, Corso Duca degli Abruzzi 24, 10129, Torino, Italy

[b] National Reference Center for Electrochemical Energy Storage (GISEL) - INSTM, Via G. Giusti 9, 50121, Firenze, Italy

[c] Luxembourg Institute of Science and Technology (LIST),5 Avenue des Hauts-Fourneaux, L-4362 Esch-sur-Alzette Luxembourg

[d] Department of Macromolecular Chemistry, Saint-Petersburg State University, Universitetsky pr. 26, 198504 Saint-Petersburg, Russia

Corresponding Authors

*E-mail: claudio.gerbaldi@polito.it (C.G.)

*E-mail: alexander.shaplov@list.lu (A.S)

Table of contents Page

**I. SYNTHESIS AND CHARACTERIZATION OF SICS**	S4
**A. Materials**	S4
**B. Synthesis**	S5
Lithium 1-[3-(methacryloyloxy)propylsulfonyl]-1-(trifluoromethanesulfonyl)imide (LiM)	S5
Ring opening polymerization (ROP) of trimethylene carbonate (TMC)	S5
RAFT synthesis of poly[TMC_n-*b*-LiM_m] block copolymers	S7
RAFT synthesis of poly[TMC_n-*b*-(LiM_m-*r*-$PEGM_k$)] block copolymers	S8
**C. Characterization**	S10
NMR and IR spectrometry	S10
Determination of number average molar mass (M_n) and monomers ratio by NMR	S10
Gel permeation chromatography (GPC)	S11
Thermal gravimetric analysis (TGA) and Differential Scanning Calorimetry (DSC)	S12
Rheology	S12
Atomic Force Microscopy (AFM)	S13
Electrochemical impedance spectroscopy (EIS)	S13
Cyclic voltammetry (CV)	S14
**D. Li cells assembly and testing**	S15
$LiFePO_4$ (LFP) based cathode preparation	S15
$LiNiMnCoO_2$ (NMC) based cathode preparation	S16
Li cells assembly	S16
Li cells testing	S16
**II. SUPPLEMENTARY TABLES AND FIGURES**	S18
**Table S1.** Ring opening polymerization (ROP) of trymethylene carbonate (TMC) using CDP RAFT-agent and DBU catalyst.	S18
**Table S2.** Li ion transference number calculated by means of Abraham's equation (Eq. 2) for poly[TMC_n-*b*-(LiM_m-*r*-$PEGM_k$)] (**copoly8**) at 70°C.	S19
**Figure S1.** Correlation of poly(TMC) experimental $\overline{M}_n$ (■, values from RI-detection, SEC in THF at 40°C) with different initiator/catalyst molar ratios.	S19
**Figure S2.** Correlation of experimental poly(TMC) $\overline{M}_n$ (■, values from RI-detection, SEC in THF at 40°C) with calculated $\overline{M}_n$ (❍) or calculated number of monomer equivalents N.	S19

Figure S3. GPC-SEC chromatograms (RI signal) of poly(TMC) 1st-block, used as a macro-chain transfer agent, and poly[TMC_n-*b*-LiM_m] (**copoly3**) and poly[TMC_n-*b*-(LiM_m-*r*-$PEGM_k$)] (**copoly8**) block copolymers after precipitation. S20
Figure S4. ^{1}H NMR of poly(TMC) (**ROP9**) (**a**), CDP RAFT agent (**b**) and TMC monomer (**c**). S21
Figure S5. ^{13}C NMR of poly(TMC) (**ROP9**). S22
Figure S6. FT-IR spectrum of poly(TMC) (**a**) and CDP RAFT agent (**b**). S23
Figure S7. ^{1}H (**a**) and ^{13}C (**b**) NMR of poly[TMC_n-*b*-LiM_m] (**copoly3**) (25 °C, DMSO-d6). S24
Figure S8. ^{19}F (**a**) and ^{7}Li (**b**) NMR of poly[TMC_n-*b*-LiM_m] (**copoly3**) (25 °C, DMSO-d6). S25
Figure S9. FT-IR spectrum of poly[TMC_n-*b*-LiM_m] (**copoly3**) (**a**) and poly[TMC_n-**b**-(LiM_m-**r**-$PEGM_k$)] (**copoly8**) (**b**). S26
Figure S10. ^{1}H NMR of poly[TMC_n-*b*-(LiM_m-*r*-$PEGM_k$)] (**copoly8**) (**a**) and poly(LiM_m-**r**-$PEGM_k$) (**b**) (25 °C, DMSO-d6). S27
Figure S11. ^{13}C NMR of poly[TMC_n-*b*-(LiM_m-*r*-$PEGM_k$)] (**copoly8**) (25 °C, DMSO-d6). S28
Figure S12. ^{19}F (**a**) and ^{7}Li (**b**) NMR of poly[TMC_n-*b*-(LiM_m-*r*-$PEGM_k$)] (**copoly8**) (25 °C, DMSO-d6). S29
Figure S13. TGA traces of poly(TMC) (**ROP9**), poly[TMC_n-*b*-(LiM_m-*r*-$PEGM_k$)] (**copoly8**), poly(PEGM) and poly(LiM) (TGA was performed on air with a heating rate of 5°C min^{-1}). S30
Figure S14. Li ion transport number analysis: Nyquist plots of the a.c. impedance of the symmetrical Li/**copoly8**/Li cell at 70°C. S30
Figure S15. Li ion transport number analysis: current variation with time during polarization in symmetrical Li/**copoly8**/Li cell at 70°C. S31
Figure S16. Interfacial stability analysis over time at 70°C of poly[TMC_n-*b*-(LiM_m-*r*-$PEGM_k$)] (**copoly8**) sandwiched in a 2 lithium metal electrode cell. S31
Figure S17. Charge/discharge potential vs specific capacity profiles at C/20 constant current rate from 4.3 to 4.8 V vs. Li+/Li with constant increment of 0.1 V each 2 cycles for Li/**copoly8**/NMC cell at 70°C. S32
Figure S18. ^{1}H (**a**), ^{13}C (**b**) and ^{19}F NMR (**c**) of LiM monomer (25 °C, DMSO-d_6). S33
Figure S19. FT-IR spectrum of LiM. S34
III. SUPPLEMENTARY REFERENCES S35

I. SYNTHESIS AND CHARACTERIZATION OF SICS

A. Materials

Poly(ethylene glycol) methyl ether methacrylate (PEGM, M_w = 500 g mol^{-1}, Aldrich), 4-cyano-4-[(dodecylsulfanylthiocarbonyl)sulfanyl]pentanol (CDP, > 97%, Aldrich), 4-methoxyphenol (MeHQ, 99%, Acros), trifluoromethanesulfonamide (97%, ABCR), lithium hydride (LiH, 97%, Aldrich), triethylamine (TEA, Et_3N, ≥ 99%, Merck), trimethylene carbonate (TMC, medical grade, Medin-N Ltd., Russia), methanol (MeOH, 99%, Acros), diethyl ether (Et_2O, 99%, Acros), ethyl acetate (EtAOc, 99%, Acros), acetonitrile (ACN, 99%, anhydrous, Acros), dimethylformamide (DMF, 99.8%, anhydrous, Acros), carbon-coated lithium iron phosphate ($LiFePO_4$, LFP, Advanced Lithium Electrochemistry Co. Ltd.), Lithium nickel manganese cobalt oxide ($LiNiMnCoO_2$, NMC, 532 BASF), carbon black C_{65} (Timcal), and lithium metal foils (Li, high purity lithium metal, Albemarle) were used as received. Dicloromethane (DCM, 99%, Acros), toluene (99%, Acros) and tetrahydrofuran (THF, 99%, Acros) were purified by solvent purification system (SPS, Mbraun). 2,2'-Azobis(isobutyronitrile) (AIBN, initiator, 98%, Acros) was recrystallized from MeOH before use. Pentane (≥99%, Aldrich) was distilled over metal Na. Thionyl chloride ($SOCl_2$, >99%, Sigma-Aldrich) and 1,8-diazabicyclo[5.4.0]undec-7-ene (DBU, 98%, Sigma-Aldrich) were purified by distillation over linseed oil and CaH_2, respectively. Benzoic acid (≥ 99.5%, Acros) was sublimed before use. Potassium 3-(methacryloyloxy)propane-1-sulfonate (98%, Aldrich) was dried at 25 °C/1 mm Hg overnight. The Spectra/Por 3 (Spectrum labs) dialysis tubing with MWCO 3500 Da was used for polymer dialysis.

B. Synthesis

Lithium 1-[3-(methacryloyloxy)propylsulfonyl]-1-(trifluoromethanesulfonyl)imide (LiM)

Lithium 1-[3-(methacryloyloxy)propylsulfonyl]-1-(trifluoromethanesulfonyl)imide was synthesized via three step procedure in a full accordance with previous reports.[1,2] The resultant crystalline powder was dried at 25 °C/1 mm Hg overnight and stored under inert atmosphere in an argon-filled glove-box (MBRAUN MB-Labstar, H_2O and O_2 content < 0.5 ppm). Spectroscopic data of the target compound were in accordance with those reported in the literature[1,2] (see **Figure S18 and S19**): ^{1}H NMR (600 MHz, DMSO-d$_6$): δ = 6.03 (s, 1H, C$\underline{H}_2$=C(CH$_3$)–), 5.67 (s, 1H, C$\underline{H}_2$=C(CH$_3$)–), 4.17 (t, 2H, CO-O-C$\underline{H}_2$-, J_{HH} = 6.4 Hz), 3.06 (m, 2H, -C$\underline{H}_2$-SO$_2$-N), 2.01 (m, 2H, O-C$\underline{H}_2$-CH$_2$-), 1.88 (s, 3H, CH$_2$=C(C$\underline{H}_3$)–); ^{13}C NMR (151 MHz, DMSO-d$_6$): δ = 166.5 (=C–$\underline{C}$O), 135.9 (CH$_2$=$\underline{C}$), 125.8 ($\underline{C}$H$_2$=C), 123.4 – 116.9 (q, J_{CF}= 324 Hz, CF$_3$), 62.8 (O$\underline{C}$H$_2$–CH$_2$–CH$_2$S), 51.3 ($\underline{C}$H$_2$S), 23.6 ($\underline{C}$H$_2$–CH$_2$S), 17.9 (=C–$\underline{C}$H$_3$); ^{19}F NMR (565 MHz, DMSO-d$_6$): δ = -79.8 (s, CF$_3$); ^{7}Li NMR (233 MHz, DMSO-d$_6$): δ = -1.02 (s); IR (ATR-mode): 2982 (w, ν_{CH}), 2935 (w, ν_{CH}), 1703 (s, $\nu_{C=O}$), 1662 (m), 1637 (m, $\nu_{C=C}$), 1463 (m), 1413 (m), 1323 (vs, ν_{asSO2}), 1267 (s, ν_{CF}), 1228 (m), 1183 (vs, ν_{sSO2}), 1108 (vs), 1068 (vs, ν_{CF}), 1023 (m), 971 (m), 940 (w), 859 (m), 822 (m), 757 (w), 717 (s), 624 (s), 587 (s) 559 (w), 510 (s) cm^{-1}.

Ring opening polymerization (ROP) of trimethylene carbonate (TMC)

Poly(TMC) precursor (macro-RAFT agent) was prepared via ring opening polymerization (ROP) using the 4-cyano-4-[(dodecylsulfanylthiocarbonyl)sulfanyl]pentanol (CDP) RAFT agent as the initiator and DBU as the catalyst. Several synthetic conditions were attempted, where the initiator to catalyst (In:Cat) ratio and the nature of solvent were varied (see **Table S1**). Typical polymerization procedure is given bellow by an example of **ROP 9** synthesis:

The synthesis was conducted in an argon-filled glove-box (MBRAUN MB-Labstar, H_2O and O_2 content < 0.5 ppm). TMC monomer (0.50 g, 4.89 mmol) and CDP RAFT agent (0.0127 g, 0.033 mmol) were dissolved in 2.00 mL of DCM in a dry Schlenk flask equipped with magnetic stirring bar. To the obtained clear monomer/initiator solution the solution of DBU catalyst (0.0248 g, 0.163 mmol) in 0.45 mL of DCM was added via syringe at 22°C (average ambient laboratory temperature) to induce the polymerization reaction. The flask was capped with a rubber septum and the stirring was continued at 22°C for 40 h. Benzoic acid (0.0299 g, 0.245 mmol, 1.5 molar equivalent relative to DBU) was added to the vial to terminate polymerization by protonating the catalyst and after stirring for 10 minutes the solution was precipitated into the excess of methanol. Polymer in a form of yellowish gummy rubber was redissolved in DCM and precipitated for the second time into the MeOH excess. The resultant polymer was collected and dried at 55 °C/1 mm Hg for 12 h. Yield: 0.41 g (82%); ^{1}H NMR (600.2 MHz, $CDCl_3$): δ = 4.28 (2H, t, *J* = 6.2 Hz, $C\underline{H}_2CH_2CH_2OH$ = TMC end group), 4.23 (660H, t, *J* = 6.3 Hz, CH_2O TMC), 3.72 (2H, t, *J* = 6.0 Hz, CH_2OH), 3.31 (2H, t, *J* = 7.5 Hz, CH_2S = Alk-1), 2.26 (1H, m, $CH\underline{H}CCN$), 2.14 (1H, m, $C\underline{H}HCCN$), 2.04 (330H, quint, *J* = 6.3 Hz, $C\underline{H}_2CH_2O$, TMC), 1.90 (2H, m, $C\underline{H}_2CH_2OH$), 1.87 (3H, s, CH_3CCN), 1.68 (2H, m, Alk-2), 1.38 (2H, m, Alk-3), 1.24 (16H, m, Alk-4,5,6,7,8,9,10,11), 0.86 (3H, t, *J* = 7.0 Hz, Alk-12); ^{13}C NMR (150.9 MHz, $CDCl_3$): δ = 217.65 (CS_3), 155.01 (CO_3), 119.14 (CN), 65.14 ($\underline{C}H_2CH_2CH_2OH$ = TMC end group), 64.39 (TMC m.u.), 59.06 ($\underline{C}H_2OH$), 44.76 ($\underline{C}$-CN), 37.13 ($\underline{C}H_2S$ = Alk-1), 35.70 ($\underline{C}H_2$-CCN), 32.00 (Alk-10), 31.75 ($C\underline{H}_2CH_2OH$), 29.71 (2C), 29.63, 29.51, 29.43 (Alk-5,6,7,8,9), 29.22 (Alk-4), 29.06 (Alk-3), 28.15 (TMC m.u.), 27.78 (Alk-2), 24.99 ($\underline{C}H_3CCN$), 24.41 ($\underline{C}H_2CH_2CCN$), 22.77 (Alk-11), 14.21 (Alk-12); IR (ATR-mode): 2975 (w, ν_{CH}), 2916 (w, ν_{CH}), 1740 (vs, $\nu_{C=O}$), 1477 (m, δ_{asCH3}), 1411 (m), 1385 (w), 1331 (m), 1310 (s), 1239 (vs, $\nu_{as\text{-}C\text{-}O\text{-}C\text{-}}$), 1136 (w), 1086 (w, $\nu_{(RS)2C=S}$), 1033 (s, $\nu_{s\text{-}C\text{-}O\text{-}C\text{-}}$), 947 (s), 924

(s), 794 (s), 722 (w, $\nu_{-(CH2)n-}$), 516 (w) cm^{-1}; $M_{n\ target}$ = 15315 g/mol; $M_{n\ SEC}$ = 16000 g/mol; M_w/M_n = 1.31 (SEC); T_g = -15°C (DSC).

RAFT synthesis of poly[TMC$_n$-*b*-LiM$_m$] block copolymers

RAFT polymerization technique was used to grow the poly(LiM) block from poly(TMC) macro-RAFT agent and, as a result, to prepare a set of poly[TMC$_n$-*b*-LiM$_m$] copolymers (Table 1, **copoly1** – **copoly3**). Typical polymerization procedure is given bellow by an example of **copoly3** (poly[TMC$_{196}$-*b*-LiM$_{13}$]) synthesis:

A solution of poly(TMC) precursor (0.56 g, M_n = 20100 g mol^{-1}, 27.78 μmol), LiM monomer (0.50 g, 1.45 mmol), and AIBN (0.91 mg, 5.56 μmol, [AIBN]:[macro-RAFT]=1:5 by mol) in anhydrous DMF (3.4 mL, 3.18 g, [DMF]:[poly(TMC)+LiM]=3:1 by weight) was transferred to a Schlenk tube equipped with magnetic stir bar and de-gassed via three freeze-pump-thaw cycles. Polymerization was further carried out under inert atmosphere at 60°C for 48 h. The resultant viscous polymer solution was diluted with DMF and precipitated in the excess of diethyl ether, whereupon it was redissolved in ethyl acetate and precipitated in the excess of diethyl ether for the second time. Isolated block copolymer represented yellow solid, and it was dried at 55°C/1 mm Hg for 12 h in B-585 oven (Buchi Glass Drying Oven, Switzerland) filled with P_2O_5. Yield: 0.71 g (67%); ^{1}H NMR (600.2 MHz, DMSO-d$_6$): δ = 4.53 (<0.1H, t, J = 4.8 Hz, OH), 4.40 (<0.1H, t, J = 5.6 Hz, $C\underline{H}_2$-CH_2-CH_2-CCN), 4.26 (0.5H, t, *J* = 6.2 Hz, $C\underline{H}_2CH_2CH_2OH$ = TMC end group), 4.14 (28H, t, *J* = 6.2 Hz, CH_2O TMC), 4.01 (2H, br. m, $COOC\underline{H}_2$), 3.45 (<0.15H, q, *J* = 6.0 Hz, $C\underline{H}_2OH$ TMC end group), 3.28 (<0.1H, br. m, Alk-1), 3.01 (2H, br. m, $C\underline{H}_2SO_2$), 2.08 (<0.1H, m, $CH\underline{H}CCN$), 2.06 (<0.1H, m, $C\underline{H}HCCN$), 2.05 (0.3H, m, $C\underline{H}_2$-CH_2CCN), 1.97 (2H, br. m, $C\underline{H}_2CH_2SO_2$), 1.94 (14H, quint, *J* = 6.2 Hz, CH_2 TMC), 2.0-1.6 (2H, br. m, $C\underline{H}_2$ methacrylate),

1.73 (0.5H, quint, *J* = 6.0 Hz, C<u>H</u>$_2$CH$_2$OH TMC end group), 1.64 (<0.1H, m, Alk-2), 1.55 (<0.1H, m, C<u>H</u>$_3$CCN CTA), 1.33 (<0.1H, m, Alk-3), 1.24 (0.4H, Alk-4,5,6,7,8,9,10,11), 0.92 & 0.75 (3H, br. m, CH$_3$ methacrylare), 0.85 (<0.1H, t, *J* = 7.0 Hz, Alk-12); ^{13}C NMR (150.9 MHz, DMSO-d$_6$): δ = 176.84 & 176.27 (CO$_2$ methacrylate), 154.42 (CO$_3$ TMC), 120.15 (q, CF$_3$, J = 325 Hz), 64.94 (weak, -<u>C</u>H$_2$OCO$_2$ TMC end group), 64.25 (CH$_2$O TMC), 63.30 (SO$_2$-CH$_2$-CH$_2$-<u>C</u>H$_2$O), 57.01 (weak, -<u>C</u>H$_2$OH TMC end group), 53.55 (br. CH$_2$ methacrylate), 51.11 (CH$_2$SO$_2$), 44.21 (<u>C</u>CH$_3$ methacrylate), 35.32 (weak, Alk-1), 31.49 (weak, <u>C</u>H$_2$CH$_2$OH TMC end group), 29.6-27.6 (weak, Alk-4-10), 27.61 (weak, Alk-2 & Alk-3), 27.52 (CH$_2$, TMC), 25.27 (weak, <u>C</u>H$_3$CCN CTA), 23.00 (<u>C</u>H$_2$CH$_2$SO$_2$), 22.13 (weak, Alk-11) 17.94 & 16.56 (CH$_3$ methacrylate), 13.67 (weak, Alk-12); ^{19}F NMR (565 MHz, DMSO-d$_6$): δ = -79.7 (s, CF$_3$); ^{7}Li NMR (233 MHz, DMSO-d$_6$): δ = 3.74 (s); IR (ATR-mode): 2973 (w, ν_{CH}), 2917 (w, ν_{CH}), 1743 (vs, $\nu_{C=O}$), 1470 (m), 1410 (m), 1330 (s, ν_{asSO2}), 1243 (vs, $\nu_{as\text{-}C\text{-}O\text{-}C\text{-}}$), 1190 (s, ν_{sSO2}), 1124 (s), 1062 (m, ν_{CF}), 1033 (s, $\nu_{s\text{-}C\text{-}O\text{-}C\text{-}}$), 972 (m), 791 (s), 715 (w), 600 (w), 572 (w), 521 (s) cm^{-1}; $M_{n\ target}$ = 38100 g/mol; $M_{n\ SEC}$ = 24400 g/mol; M_w/M_n = 1.36 (SEC); T_{g1} = -14°C, T_{g2} = 140°C (DSC); T_{onset} = 205°C (TGA).

RAFT synthesis of poly[TMC$_n$-b-(LiM$_m$-r-PEGM$_k$)] block copolymers

Random RAFT copolymerization was performed to prepare a set of poly[TMC$_n$-*b*-(LiM$_m$-*r*-PEGM$_k$)] block copolymers starting from the poly(TMC) macro-RAFT agent with fixed length (**Table 2, copolymer 4 – copolymer 8**). Typical polymerization procedure is given bellow by an example of **copoly8** (poly[TMC$_{196}$-*b*-(LiM$_4$-*r*-PEGM$_{31}$)]) synthesis:

A solution of poly(TMC) precursor (3.39 g, M_n = 20100 g mol^{-1}, 0.17 mmol), LiM monomer (0.62 g, 1.78 mmol), PEGM (4.46 g, 8.91 mmol) and AIBN (5.55 mg, 33.81 μmol, [AIBN]:[macro-RAFT]=1:5) in anhydrous DMF (71.5 mL, 67.8 g, [DMF]:[poly(TMC)+ILM+PEGM]=8:1 by

weight) was transferred to a Schlenk tube equipped with magnetic stir bar and de-gassed via three freeze-pump-thaw cycles. Polymerization was further carried out under inert atmosphere at 60°C for 48 h. The resultant viscous polymer solution was precipitated in the excess of diethyl ether, redissolved in ethyl acetate and again reprecipitated in the excess of diethyl ether. Isolated block copolymer represented yellow rubber-like material, and it was dried at 55°C/1 mm Hg for 12 h in B-585 oven (Buchi Glass Drying Oven, Switzerland) filled with P_2O_5. Yield: 7.85 g (93%); ^{1}H NMR (600.2 MHz, DMSO-d_6): δ = 4.54 (<0.1H, t, J = 4.8 Hz, OH), 4.26 (0.2H, t, *J* = 6.2 Hz, $C\underline{H}_2CH_2CH_2OH$ = TMC end group), 4.14 (25H, t, J = 6.0 Hz, CH_2O TMC), 4.02 (1.5H, m, $COOC\underline{H}_2$), 3.61-3.52 (29H, br. m, $OC\underline{H}_2C\underline{H}_2O$), 3.44 (0.2H, m, $C\underline{H}_2OH$ TMC end group), 3.25 (2.8H, s, $C\underline{H}_3O$), 3.01 (0.2H, br. m, $C\underline{H}_2SO_2$), 2.05 (0.15H, m, $C\underline{H}_2$-CH_2CCN), 1.94 (12 H, quint, J = 6.0 Hz, $C\underline{H}_2$ TMC), 2.1-1.6 (0.2H, br. m, $C\underline{H}_2CH_2SO_2$), 1.89-1.84 (2H, br. m, $C\underline{H}_2$ methacrylate), 1.73 (0.4H, m, $C\underline{H}_2CH_2OH$ end group), 1.30 (<0.1H, m, Alk-3), 1.24 (0.4H, Alk-4,5,6,7,8,9,10,11), 0.95 & 0.79 (3H, br. m, $C\underline{H}_3$ methacrylare), 0.86 ((<0.1H, m, Alk-12); ^{13}C NMR (150.9 MHz, DMSO-d_6): δ = 176.78 &176.04 (CO_2), 154.37 (CO_3), 121.13 (q, *J* = 324 Hz), 71.28 ($\underline{C}H_2OCH_3$ end group), 69.79 ($\underline{C}H_2\underline{C}H_2O$ ethylene glycol units), 69.59 ($\underline{C}H_2CH_2OCH_3$ end group), 67.82 ($COO\underline{C}H_2CH_2O$), 64.89 (weak, -$\underline{C}H_2CH_2CH_2OH$ TMC end group), 64.19 ($\underline{C}H_2O$ TMC), 63.22 ($O\underline{C}H_2CH_2CH_2SO_2$) 58.01 ($O\underline{C}H_3$), 56.94 ($\underline{C}H_2OH$ TMC end group), 53.9 (br. CH_2 methacrylate), 51.13 ($\underline{C}H_2SO_2$), 44.54 & 44.17 (br., $\underline{C}CH_3$ methacrylate), 31.42 ($\underline{C}H_2CH_2OH$ TMC end group), 28.99-28.41 (weak, Alk-4-10), 27.48 ($\underline{C}H_2$ TMC), 23.07 ($\underline{C}H_2CH_2SO_2$), 18.11 & 16.39 (CH_3 methacrylate) 13.86 (weak, Alk-12); ^{19}F NMR (565 MHz, DMSO-d_6): δ = -79.7 (s, CF_3); ^{7}Li NMR (233 MHz, DMSO-d_6): δ = -0.98 (s); IR (ATR-mode): 2960 (w, ν_{CH}), 2878 (w, ν_{CH}), 1742 (vs, $\nu_{C=O}$), 1470 (m), 1407 (m), 1330 (s, ν_{asSO2}), 1244 (vs, $\nu_{as\text{-}C\text{-}O\text{-}C\text{-}}$), 1182 (s, ν_{sSO2}), 1119 (vs, $\nu_{\text{-}C\text{-}O\text{-}}$), 1034 (vs, $\nu_{s\text{-}C\text{-}O\text{-}C\text{-}}$), 933 (s), 862 (w, $\nu_{s\text{-}CH2\text{-}CO\text{-}}$), 791 (s), 620 (w), 579 (w), 535

(w), 514 (w) cm^{-1}; Anal. Calcd. for poly[TMC_{196}-b-(LiM_4-r-$PEGM_{31}$)] $C_{1478}H_{2479}F_{12}Li_4N_4O_{922}S_{11}$ (35666.0): C, 49.76%; H, 7.00%; N, 0.16%. Found: C, 49.14%; H, 6.95%; N, 0.18%; $M_{n\ target}$ = 50100 g/mol; $M_{n\ SEC}$ = 34600 g/mol; M_w/M_n = 1.39 (SEC); T_{g1} = -51ºC, T_{g1} = -16ºC (DSC); T_{onset} = 155ºC (TGA).

C. Characterization

NMR and IR spectrometry

NMR spectra were recorded on Avance III HD 600 MHz spectrometer (Bruker) at 25 °C in the indicated deuterated solvents and are listed in ppm. The signal corresponding to the residual protons of the deuterated solvent was used as an internal standard for ^{1}H and ^{13}C NMR, while the C_6F_6 and $LiBF_4$ were utilized as external standards for ^{19}F and ^{7}Li, respectfully. Signal assignment was performed using 2D NMR techniques: heteronuclear single quantum coherence (HSQC), and heteronuclear multiple bond correlation (HMBC). IR spectra were acquired on a Nicolet Magna-750 Fourier IR-spectrometer using KBr pellets or on Brucker Tensor 27 Fourier IR-spectrometer using ATR technology (128 scans, resolution is 2 cm^{-1}) and Spectragryph optical spectroscopy software.

Determination of number average molar mass (M_n) and monomers ratio by NMR

^{1}H NMR in deuterated chloroform was used to determine the experimental molar mass (M_{NMR}) of poly(TMC). The M_{NMR} of poly(TMC) was defined by simplified equation 1:

$$M_{NMR} = N_{PC} \cdot M_{PC} \qquad \text{Eq. 1,}$$

where N_{PC} – number of PC monomer units, which is determined by number of protons under **11**/**2** (Figure S4a), when number of protons under **1** (Figure S4a) is set to 3; M_{PC} – molar mass of PC unit (M_{PC} = 102.1 g mol^{-1}).

^{1}H NMR in deuterated DMSO was used to determine the experimental molar ratio for PEGM and LIM blocks in poly[TMC_n-*b*-(LiM_m-*r*-$PEGM_k$)]. The PEGM:LiM molar ratio was defined by equation 2:

$$\frac{PEGM}{LiM} = \frac{1}{N_{LiM}/_2} \quad \text{Eq. 2,}$$

where N_{LiM} – number of protons under **7** (Figure S10b), when number of protons under **10** (Figure S10b) is set to 3.

Gel permeation chromatography (GPC)

For determination of M_n, M_w and M_w/M_n of poly(TMC) macromolecules a 1260 Infinity II gel permeation chromatograph (Agilent Technologies) was used. The chromatograph was equipped with an integrated multi-detector composed of IR, UV, viscometer and light scattering. The separation was conducted using in series a PLgel guard column, PLgel 5 mm MIXED-D column, and PLgel 5 mm MIXED-C column (Agilent Technologies). The eluent was THF and the flow rate was of 1.0 mL·min^{-1} at 40 ºC. In the same time, for measuring of M_n, M_w and M_w/M_n for ionic poly[TMC_n-*b*-LiM_m] and poly[TMC_n-*b*-(LiM_m-*r*-$PEGM_k$)] block copolymers a 1200 Infinity gel permeation chromatograph (GPC, Agilent Technologies) was applied. The chromatograph was equipped with an integrated IR detector, a PL PolarGel-M column and a PL PolarGel-M guard column (Agilent Technologies). 0.1 M solution of $Li(CF_3SO_2)_2N$ in DMF was used as an eluent, the flow rate was maintained at 1.0 mL·min^{-1} and the measurements were performed at 50°C. For all polymers the polymethylmethacrylate standards (EasiVial PM, Agilent Technologies, M_p = 550 - 1558×10^3) were used to perform calibration.

Degree of polymerization (DP) was determined using the following equation (Eq. 3):

$$DP = \frac{M_n GPC}{M_{monomer}} \quad \text{Eq. 3}$$

where $M_{n\ GPC}$ is the molar mass of the polymer determined by GPC, while $M_{monomer}$ - molar mass of the monomer.

Thermal gravimetric analysis (TGA) and Differential Scanning Calorimetry (DSC)

TGA was carried out in air on a TGA2 STAR^e System (Mettler Toledo) applying a heating rate of 5 °C·min^{-1}. For DSC measurements, all polymer samples were preliminary dried at 60 °C/1 mm Hg for 12 h in the B-585 oven (Buchi Glass Drying Oven, Switzerland) filled with P_2O_5 and were transferred under vacuum inside an argon-filled glovebox (MBRAUN MB-Labstar, H_2O and O_2 content <0.5 ppm), where they were hermetically sealed in Al pans. Prior to measurements, the calorimeter was calibrated using the indium calibration standard (Mettler-Toledo, purity > 99.999 %). DSC of polymer samples was performed on a DSC3$^+$ STAR^e System (Mettler Toledo) with a heating rate of 5 °C·min^{-1} (1st cycle) and 10 °C ·min^{-1} (2nd cycle) in the range of -80 to 80 °C. The glass transition temperature (T_g) was determined during the second heating cycle as the first one was used to eliminate the thermal history of the sample.

Rheology

Rheological measurements were performed using an Anton Paar Physica MCR 302 rheometer equipped with a CTD 450 temperature control device with a disposable aluminum parallel plate-plate (diameter: 25 mm, measure gap: 0.35 mm) geometry. Copoly8 and poly[LiM$_m$-***r***-PEGM$_k$] samples were loaded directly onto the aluminum plate of the rheometer and special care was taken to exclude bubbles. Measurements were recorded in the oscillation mode at an imposed 1% strain amplitude (γ), ensuring that both moduli G′ and G″ were obtained in the linear viscoelastic regime. All measurements were carried out at 25 and 70°C. Tests were repeated at least twice to insure good repeatability of the results.

Atomic Force Microscopy (AFM)

AFM-images were recorded with MFP-3D infinity microscope (Asylum Instruments/Oxford Instruments) in the tapping mode (-20°C, in air). AC160TS-R3 (Olympus) cantilevers were applied with a stiffness of 26 N m^{-1} and resonance frequency of 300 KHz. The domains periodicity was evaluated from 3 different 1x1μm^2 images. On each image two profiles were taken and for each the distance over 10 consecutives periods was recorded. The images were recorded in the so-called 'soft tapping mode', to avoid deformation and indentation of the polymer surface by the tip. All the images were collected with the maximum available number of pixels (512) in each direction. General procedure for the preparation of the samples for AFM was as follow: films were cast from 10 wt% solution of **copoly8** in DMF on a microscope glass slide, and allowed to slowly evaporate at 80°C. The obtained thin films were dried at 80°C/1 mm Hg for 24 h.

Electrochemical impedance spectroscopy (EIS)

Ionic conductivity (σ) was determined by electrochemical impedance spectroscopy (EIS) with a VSP potentiostat/galvanostat (Bio-Logic Science Instruments). To avoid any influence of moisture/humidity on the conductivity of polymer electrolytes, the latter were preliminary dried at 60 °C/1 mm Hg for 12 h in the B-585 oven (Buchi Glass Drying Oven, Switzerland) filled with P_2O_5 and were transferred under vacuum inside an argon-filled glovebox (MBRAUN MB-Labstar, H_2O and O_2 content <0.5 ppm). Polymers were sandwiched between two stainless steel (SS-316) blocking electrodes. The distance between the electrodes (d) was kept equal to 250 μm using a Teflon spacer ring with the inner area (A) of 0.502 cm^2. Symmetrical stainless steel/**copolymer**/stainless steel assembly was clamped into the 2032 coin cell and afterwards was taken out from glovebox. Cell impedance was measured at the open circuit potential (OCV) by applying a 50 mV perturbation in the frequency range from 10^{-2} to 2×10^5 Hz and in a temperature

range from 20 to 100 °C. Temperature was controlled using the programmed M-53 oven (Binder, Germany), where cells were allowed to reach thermal equilibrium for at least 1h before each test. The ohmic resistance (R, Ω) of the polymer electrolyte sample, obtained from the Nyquist plot at the low frequency end of the semicircle, was used to calculate the ionic conductivity using the following equation (Eq. 4):

$$\sigma = \frac{d}{A \cdot R} \quad \text{Eq. 4}$$

Cyclic voltammetry (CV)

CV was used to determine the electrochemical stability window (ESW) of solid polymer electrolytes at 70 °C. VMP3 multipotentiostat (20 V, ±400 mA, Bio-Logic Science Instruments) and ECC-Std test cells (EL-Cell GmbH, Germany) were used to carry out the electrochemical characterization. Moisture contaminations were avoided by assembling the cells inside the Ar-filled glove-box (MBraun UNILab, H_2O and O_2 content <0.1 ppm). The two-electrode cells were assembled by sandwiching copolymers between working electrode and a lithium metal foil, which served as the reference and the counter electrode, simultaneously. Separate tests were performed for the determination of the cathodic and anodic electrochemical stability. Carbon-coated aluminum and copper disks were used as working electrodes during anodic and cathodic scans, respectively. To evaluate anodic limits, potential sweeps were carried out between OCV and 5.5 V vs. Li^+/Li at a constant rate of 0.1 mV s^{-1}. To determine cathodic limits, potential sweeps were performed between OCV and -0.5 V vs. Li^+/Li at the same constant rate.

The lithium-ion transference number (t_{Li}^+) was determined at 70 °C in a symmetric lithium metal/**copolymer**/lithium metal cell, which was subjected to a 120 mV polarization bias (ΔV) with the aim to determine the initial (I_o) and the steady state (I_{ss}) currents. EIS was performed on VMP3

multipotentiostat (20 V, ±400 mA, Bio-Logic Science Instruments) by applying a 50 mV perturbation between 300 kHz and 0.5 mHz at OCV conditions to obtain the resistance of the passivation layer before ($R_{SEI+CT,o}$) and after ($R_{SEI+CT,ss}$) polarization. The t_{Li}^{+} was calculated using an Abrahams equation[3] (Eq. 5), which is the slightly modified version of the known equation proposed by Evans/Vincent/Bruce[4]:

$$t_{Li+} = \frac{I_{ss} \cdot R_{b,ss} \cdot (\Delta V - I_o \cdot R_{SEI+CT,o})}{I_o \cdot R_{b,o} \cdot (\Delta V - I_{ss} \cdot R_{SEI+CT,ss})} \qquad \text{Eq. 5}$$

, where t_{Li+} is the Li transference number, ΔV is the potential applied across the cell, $R_{SEI+CT,o}$ and $R_{SEI+CT,ss}$ are the initial and steady-state resistances of the passivating layer, I_o and I_{ss} are the initial and steady-state currents, $R_{b,o}$ and $R_{b,ss}$ are the variation of bulk electrolyte resistance.

D. Li cells assembly and testing

$LiFePO_4$ (LFP) based cathode preparation

A composition of 60 wt.% of carbon coated $LiFePO_4$, 10 wt.% of C_{65} carbon black and 30 wt.% of **copoly8** was used for the cathode preparation. Firstly, LFP active material powder and C_{65} carbon black were gently mixed in a hand mortar and, successively added to the ca. 5 wt.% solution of **copoly8** in NMP upon stirring. The stirring was continued at ambient temperature for 1 h, whereupon the resultant suspension was additionally homogenized using an Ultra-Turrax® mixer (IKA-Werke GmbH & Co. KG) for 10 min. The obtained dense slurry was casted onto a carbon coated aluminum current collector using a doctor-blade with a blade height of 300 μm. NMP solvent was removed by evaporation at ambient temperature for 12 h and further drying at 60 °C/1 mm Hg for 24 h in the B-585 oven (Buchi Glass Drying Oven) filled with P_2O_5. Without connection to atmosphere the cathode tape was further transferred under vacuum inside an argon-

filled glovebox (MBRAUN MB-Labstar, H_2O and O_2 content <0.5 ppm). The obtained composite cathode was characterized by film's thickness after drying of 60±2 μm and active mass loading of 4.17 mg cm^{-2}.

$LiNiMnCoO_2$ (NMC) based cathode preparation

NMC based cathode film was prepared following the synthetic procedure described above for LFP catholyte, however, with the exception in the composition, which was as follows: 75 wt.% of $LiNiMnCoO_2$ active material, 10 wt % of C_{65} carbon black and 15 wt.% of **copoly8**. The obtained composite cathode was characterized by film's thickness after drying of 65±2 μm and active mass loading of 4.32 mg cm^{-2}.

Li cells assembly

Lab-scale $LiFePO_4$/**copoly8**/Li and $LiNiMnCoO_2$/**copoly8**/Li battery prototypes assembly was performed inside the Ar-filled glovebox using the ECC-Std test cells (EL-Cell GmbH). A 100 μm thick polyethylene terephthalate (Mylar®) round spacer with a 10 mm internal diameter was layered on top of the composite cathode tape. Afterwards a layer of **copoly8** electrolyte was applied manually directly on the composite cathode's surface within the internal diameter of the spacer. The assembly was completed with a lithium metal disk anode.

Li cells testing

Lab-scale $LiFePO_4$/**copoly8**/Li cells were galvanostatically cycled on a VMP3 multipotentiostat (20 V, ±400 mA, BioLogic Science Instruments) at 70 °C between 2.5 and 3.8 V vs. Li^+/Li at fixed charge/discharge current regime of C/20, corresponding to a full discharge or full charge of the theoretical cathode capacity (170 mAh g^{-1}) in 20 hours.

Lab-scale $LiNiMnCoO_2$/**copoly8**/Li cells were cycled at 70 °C between 3 and 4.3 V vs. Li^+/Li at fixed charge/discharge current regime of C/20, corresponding to a full discharge or full charge of the theoretical cathode capacity (185 mAh g^{-1}) in 20 hours.

Cycling tests were also conducted at higher current rates, where the rate is denoted as C/n, corresponding here to a full discharge or full charge of the theoretical cathode capacity (C) in n hours.

II. SUPPLEMENTARY TABLES AND FIGURES

Table S1. Ring opening polymerization (ROP) of trymethylene carbonate (TMC) using CDP RAFT-agent and DBU catalyst ([TMC] = 2M, temperature - 22 °C, duration - 40 h).

Sample	$\frac{[\text{TMC}]}{[\text{Initiator}]}$ molar ratio	$\frac{[\text{Initiator}]}{[\text{Catalyst}]}$ molar ratio	Solvent	M_n (target) (g mol^{-1})	$M_{n\ (SEC)}$ (g mol^{-1}) [a]	M_w/M_n SEC [a]	Yield (%)
**ROP 1**	200:1	0.05:1	DCM	20420	11300	1.68	84
**ROP 2**	200:1	0.10:1	DCM	20420	13500	1.39	90
**ROP 3**	200:1	0.20:1	DCM	20420	16400	1.27	82
**ROP 4**	200:1	0.25:1	DCM	20420	15300	1.40	84
**ROP 5**	200:1	0.30:1	DCM	20420	12300	1.29	90
**ROP 6**	200:1	1:1	DCM	20420	9600	1.17	80
**ROP 7**	50:1	0.20:1	DCM	5105	8000	1.34	97
**ROP 8**	100:1	0.20:1	DCM	10210	12200	1.32	78
**ROP 9**	150:1	0.20:1	DCM	15315	16000	1.31	82
**ROP 10**	300:1	0.20:1	DCM	30630	14300	1.40	84
**ROP11**	150:1	0.20:1	THF	15315	10200	1.23	50
**ROP12**	150:1	0.20:1	Toluene	15315	17900	1.55	15

[a] By GPC in THF at 40°C with PMMA standards calibration.

Table S2. Li ion transference number calculated by means of Abraham's equation (Eq. 2) for poly[TMC_n-*b*-(LiM_m-*r*-$PEGM_k$)] (**copoly8**) at 70ºC.

	R_{bulk} (Ω)	R_{ct+SEI} (Ω)	I (μA)	t_{Li^+}
Initial state	14912	592	7.66	0.90
Steady state	14762	387	7.08	

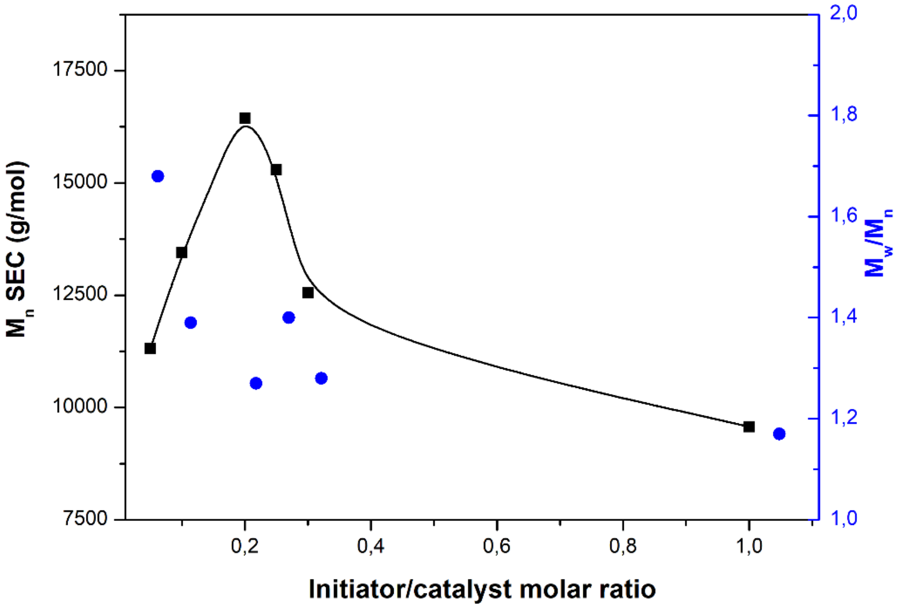

Figure S1. Correlation of poly(TMC) experimental $\overline{M}_n$ (■, values from RI-detection, SEC in THF at 40ºC) with different initiator/catalyst molar ratios.

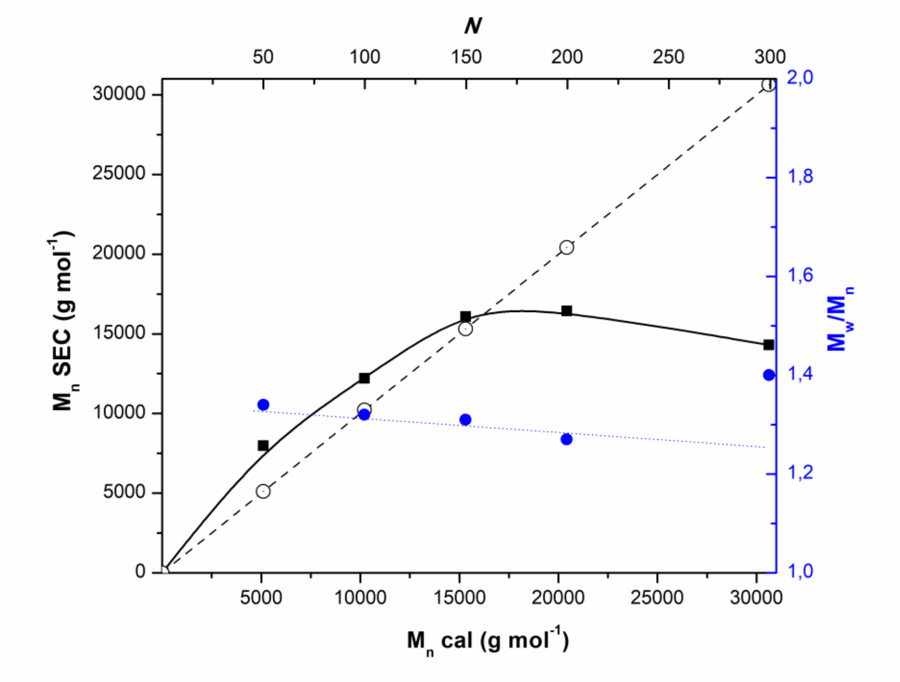

Figure S2. Correlation of experimental poly(TMC) $\overline{M}_n$ (■, values from RI-detection, SEC in THF at 40°C) with calculated $\overline{M}_n$ (❍) or calculated number of monomer equivalents N.

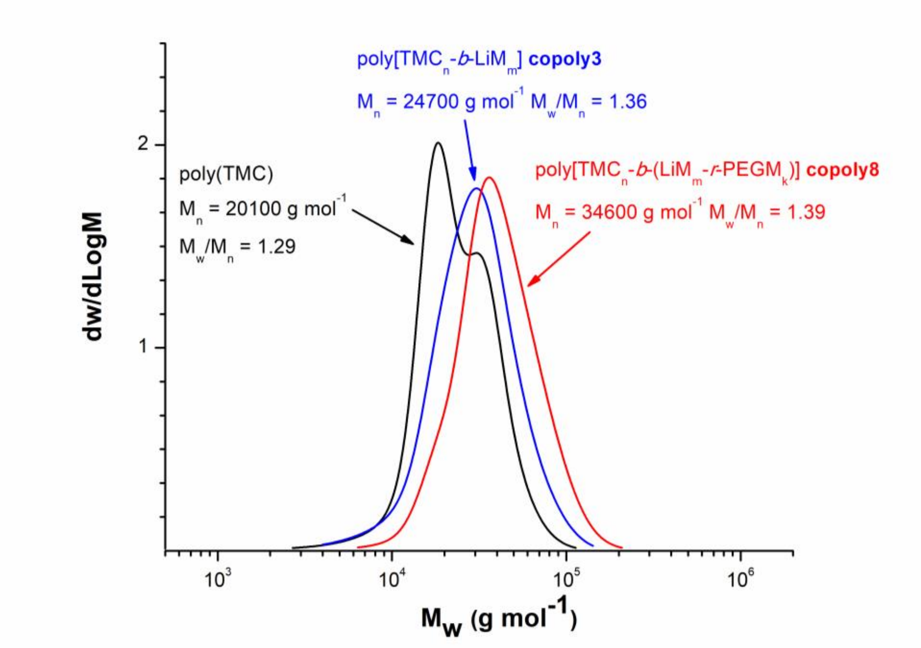

Figure S3. GPC-SEC chromatograms (RI signal) of poly(TMC) 1st-block, used as a macro-chain transfer agent, and poly[TMC_n-*b*-LiM_m] (**copoly3**) and poly[TMC_n-*b*-(LiM_m-*r*-$PEGM_k$)] (**copoly8**) block copolymers after precipitation.

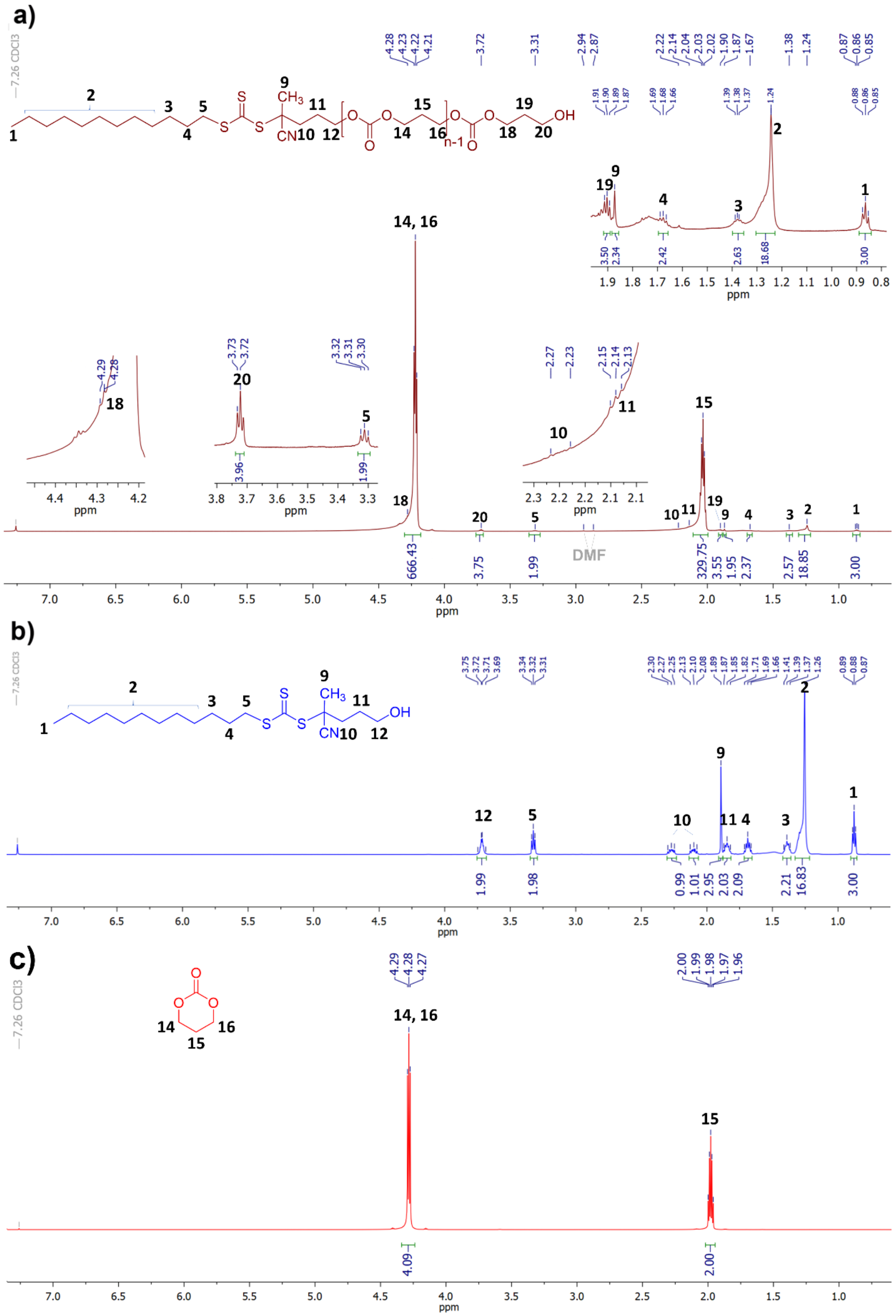

Figure S4. ^{1}H NMR of poly(TMC) (**ROP9**) (**a**), CDP RAFT agent (**b**) and TMC monomer (**c**).

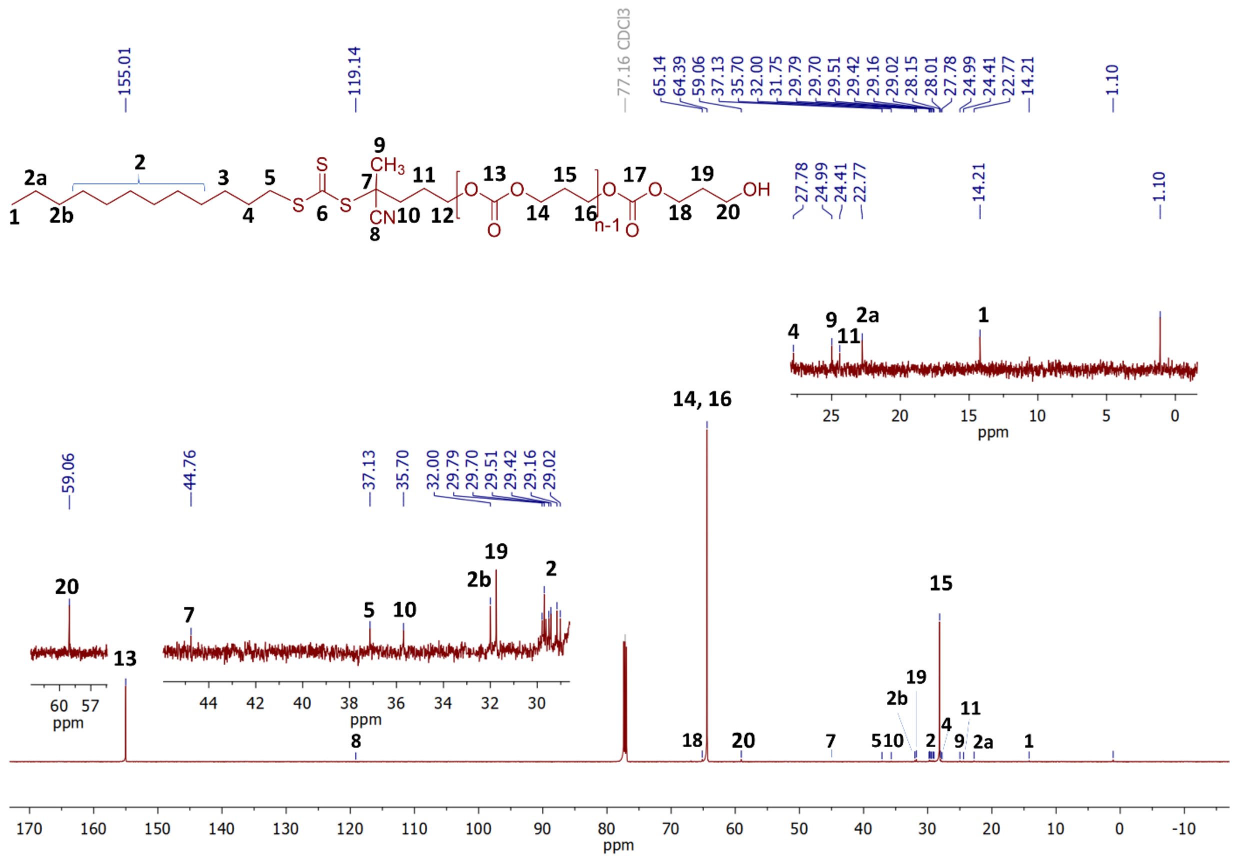

Figure S5. ^{13}C NMR of poly(TMC) (**ROP9**).

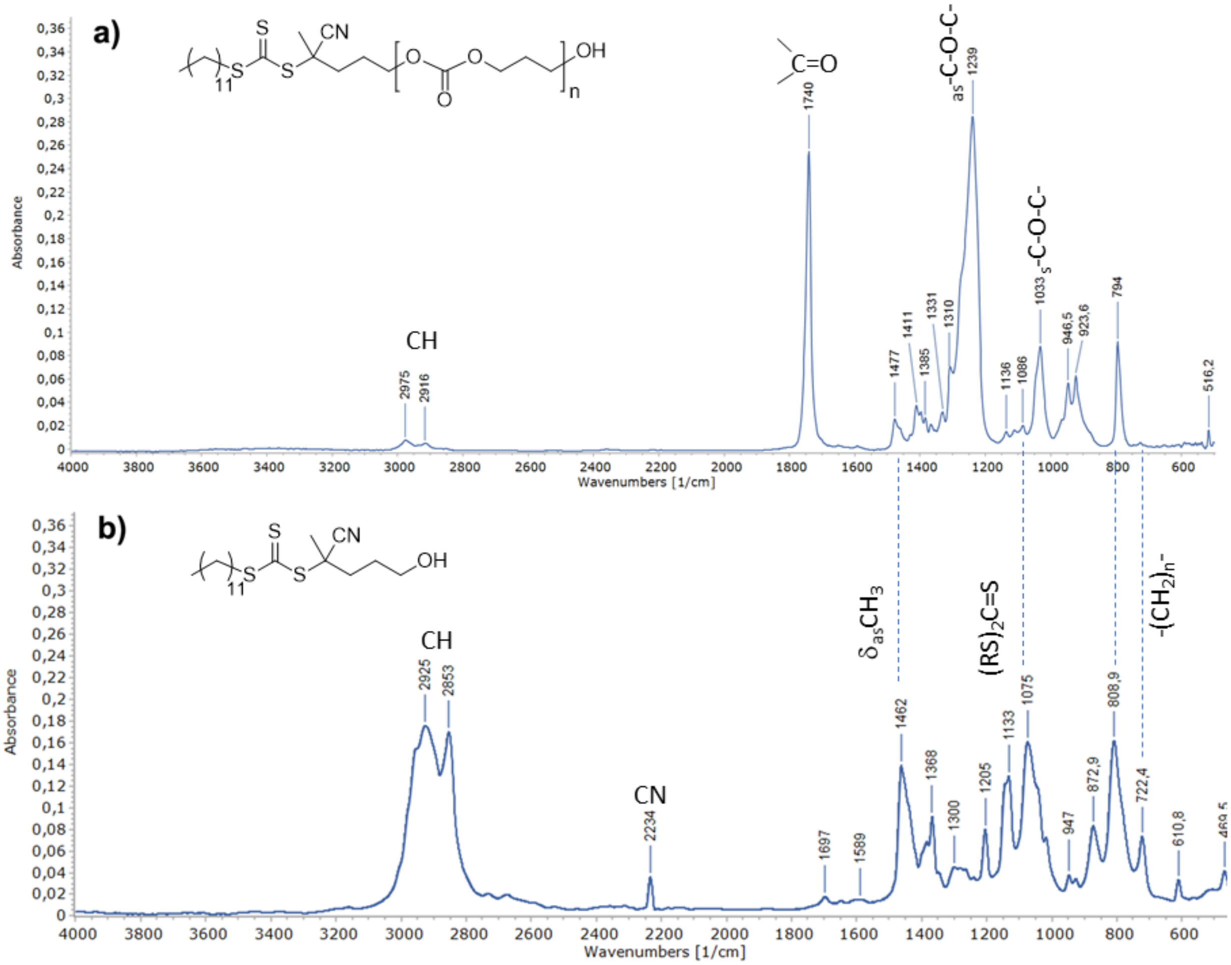

Figure S6. FT-IR spectrum of poly(TMC) (**a**) and CDP RAFT agent (**b**).

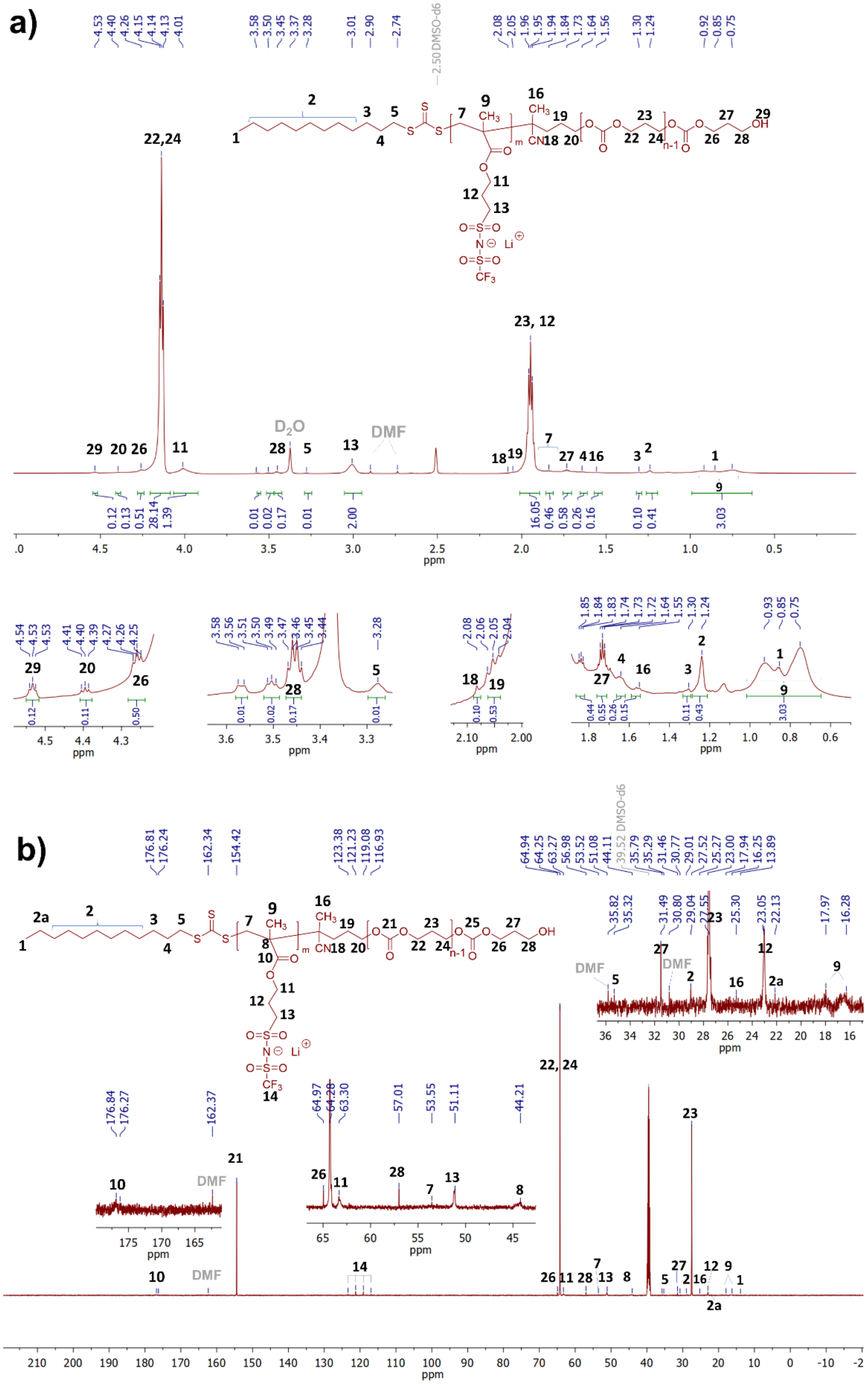

Figure S7. ^{1}H (**a**) and ^{13}C (**b**) NMR of poly[TMC_n-*b*-LiM_m] (**copoly3**) (25 °C, DMSO-d6).

a)

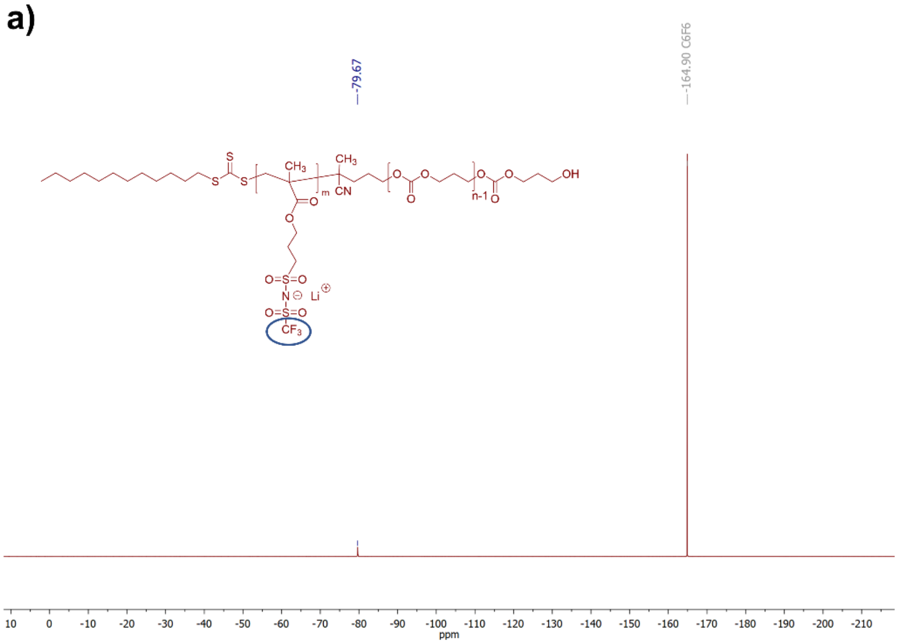

b)

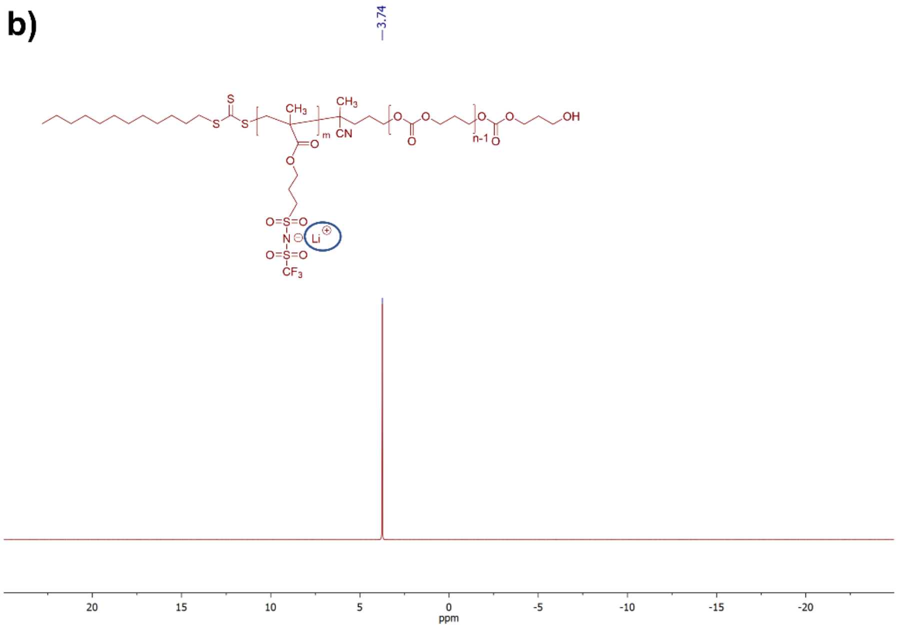

Figure S8. ^{19}F (**a**) and ^{7}Li (**b**) NMR of poly[TMC_n-*b*-LiM_m] (**copoly3**) (25 °C, DMSO-d6).

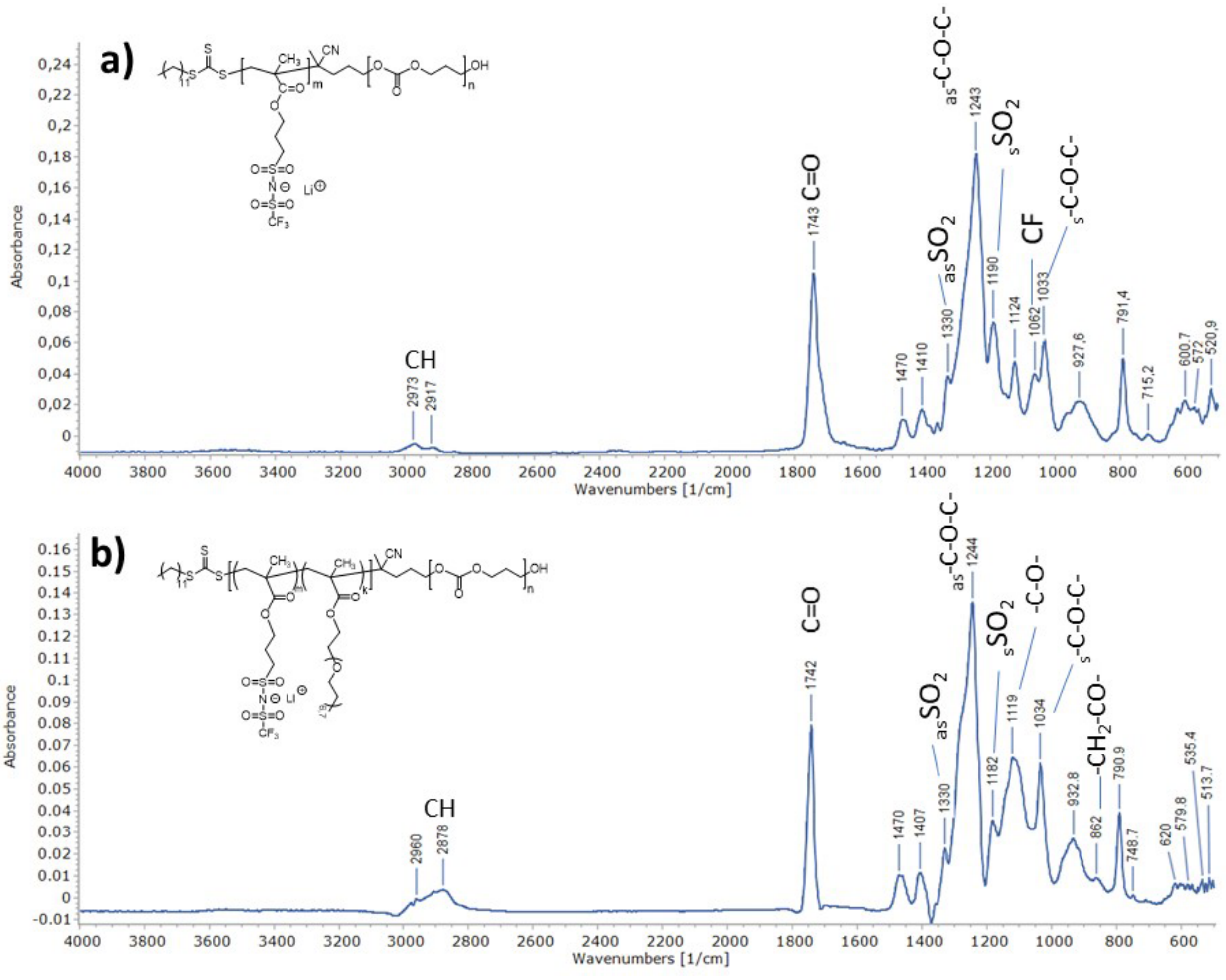

Figure S9. FT-IR spectrum of poly[TMC_n-**b**-LiM_m] (**copoly3**) (**a**) and poly[TMC_n-*b*-(LiM_m-*r*-$PEGM_k$)] (**copoly8**) (**b**).

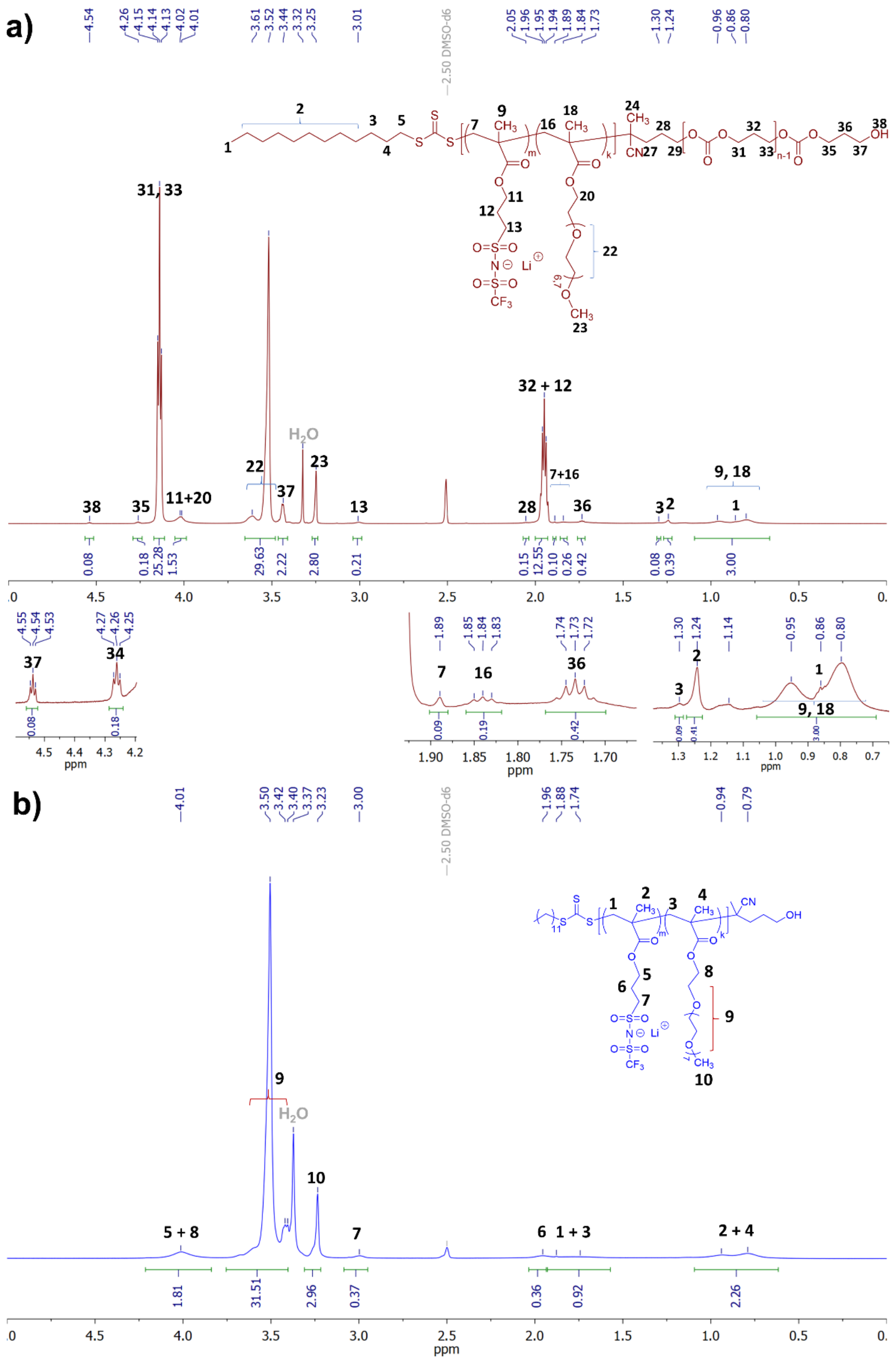

Figure S10. ^{1}H NMR of poly[TMC$_n$-*b*-(LiM$_m$-*r*-PEGM$_k$)] (**copoly8**) (**a**) and poly(LiM$_m$-*r*-PEGM$_k$) (**b**) (25 °C, DMSO-d6).

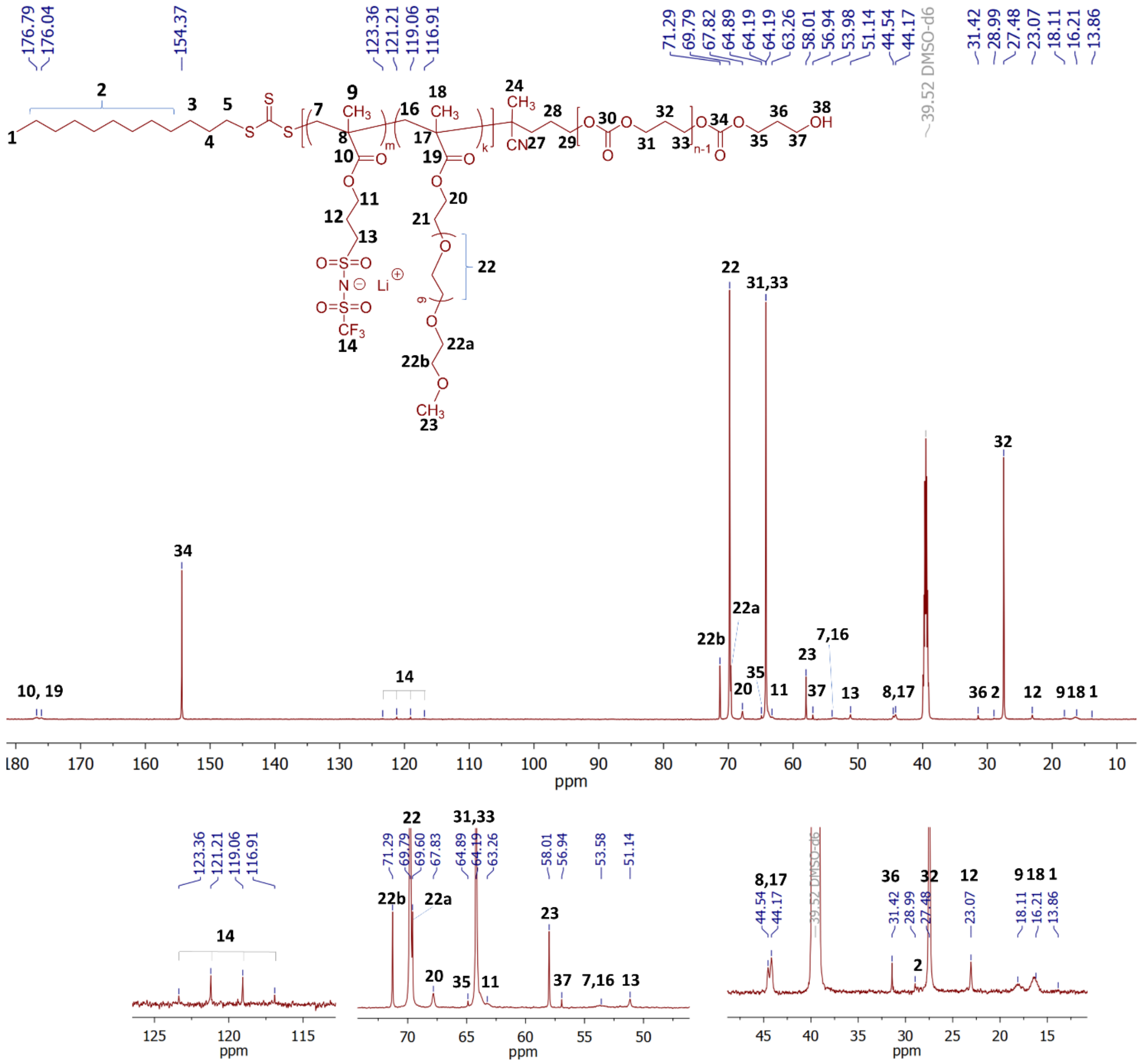

Figure S11. ^{13}C NMR of poly[TMC$_n$-*b*-(LiM$_m$-*r*-PEGM$_k$)] (**copoly8**) (25 °C, DMSO-d6).

a)

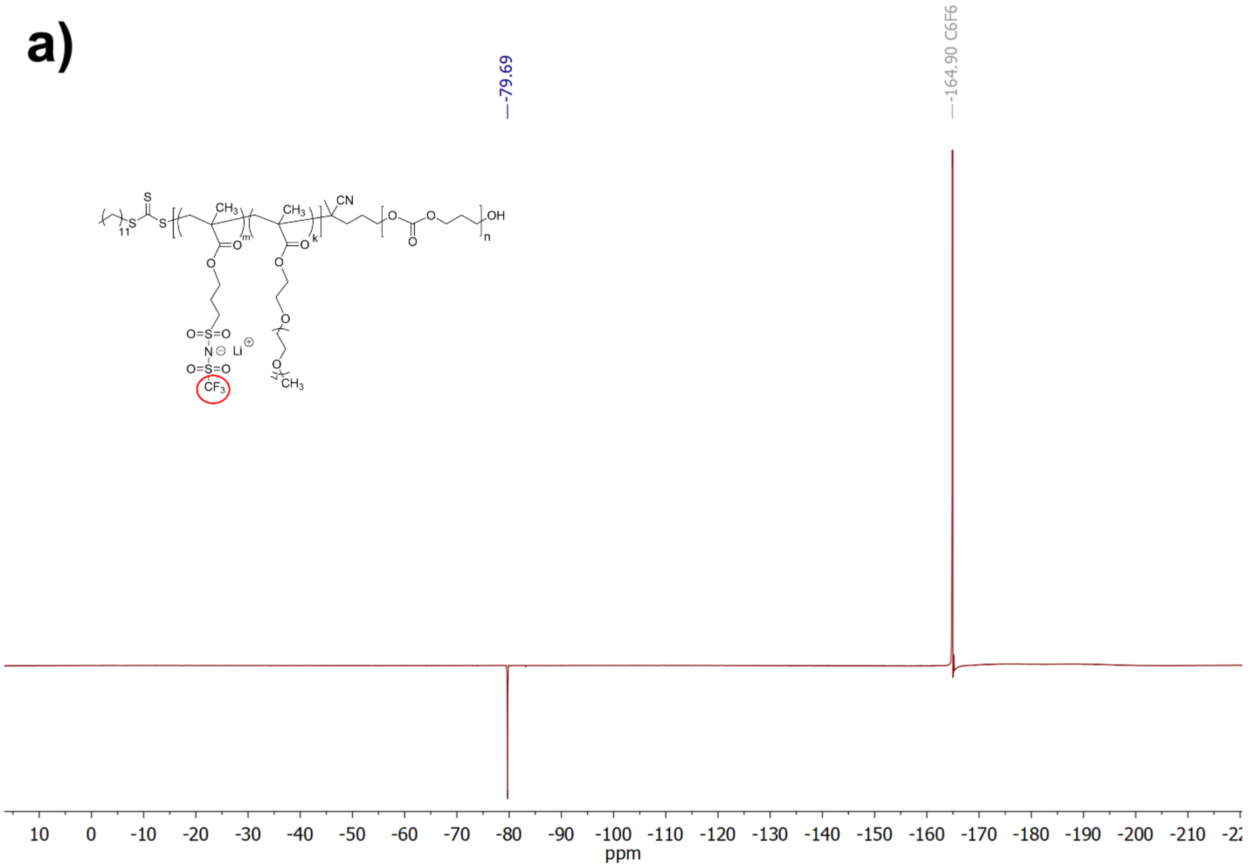

b)

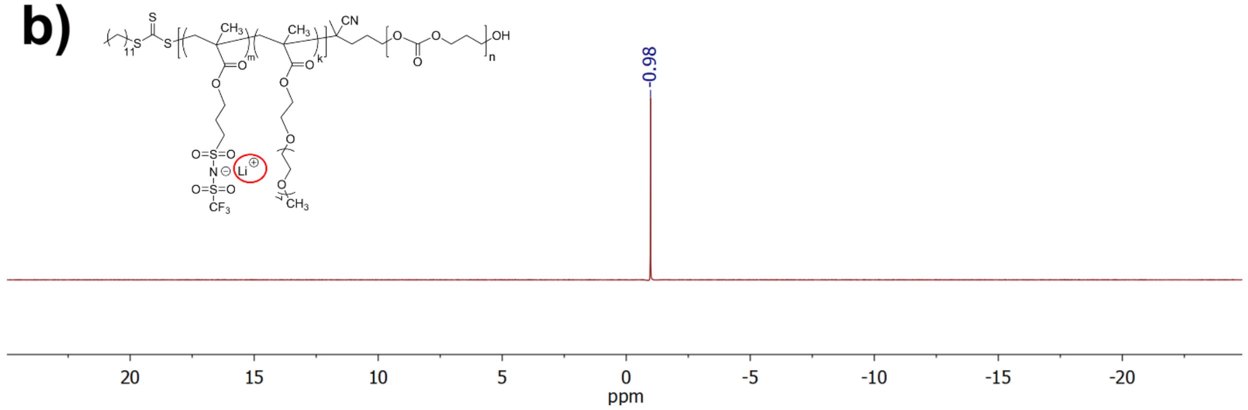

Figure S12. ^{19}F (**a**) and ^{7}Li (**b**) NMR of poly[TMC_n-*b*-(LiM_m-*r*-$PEGM_k$)] (**copoly8**) (25 °C, DMSO-d6).

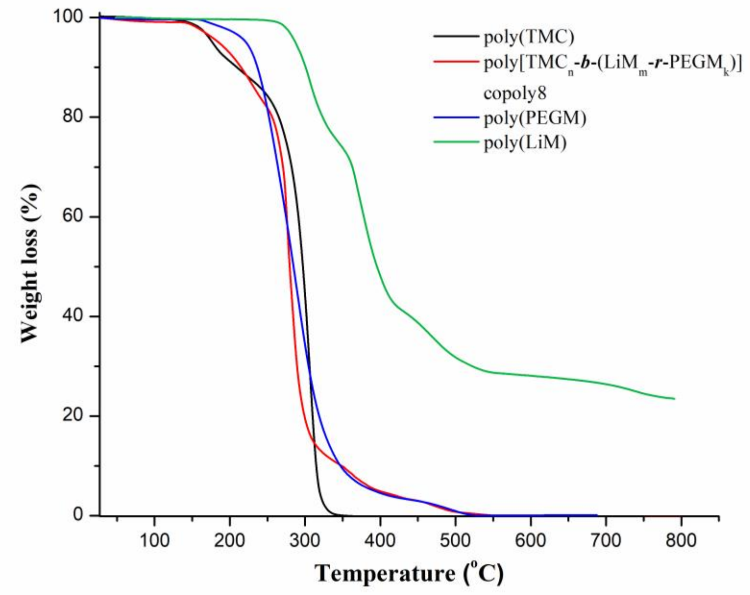

Figure S13. TGA traces of poly(TMC) (**ROP9**), poly[TMC_n-*b*-(LiM_m-*r*-$PEGM_k$)] (**copoly8**), poly(PEGM) and poly(LiM) (TGA was performed on air with a heating rate of 5°C min^{-1}).

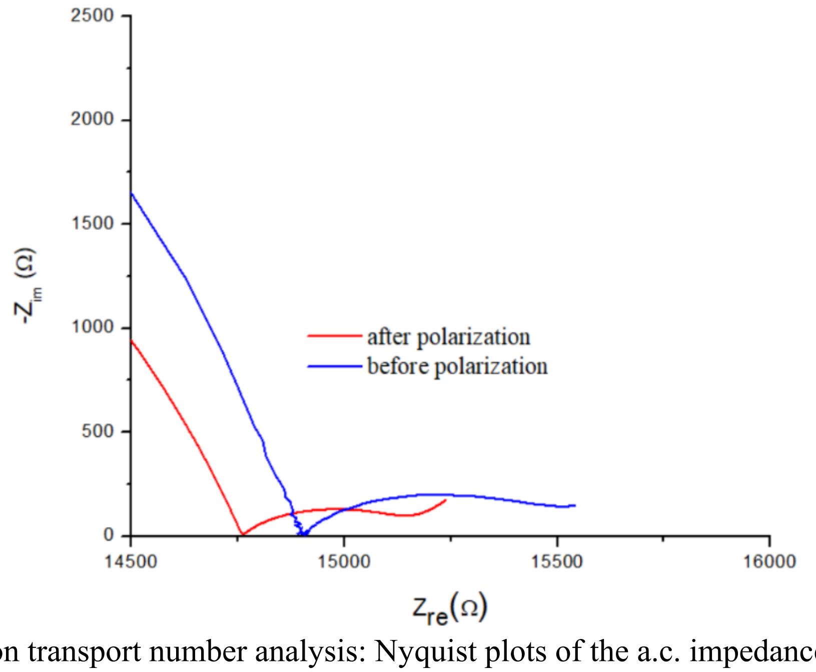

Figure S14. Li ion transport number analysis: Nyquist plots of the a.c. impedance of the symmetrical Li/**copoly8**/Li cell at 70°C.

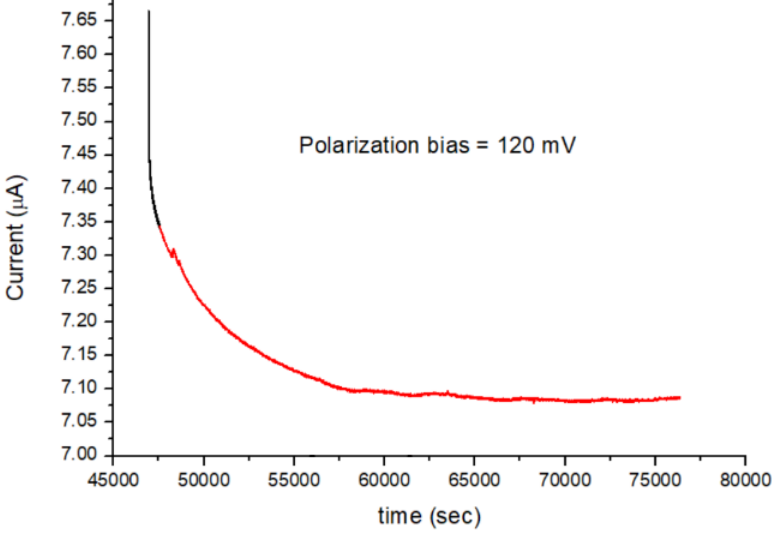

Figure S15. Li ion transport number analysis: current variation with time during polarization in symmetrical Li/**copoly8**/Li cell at 70ºC.

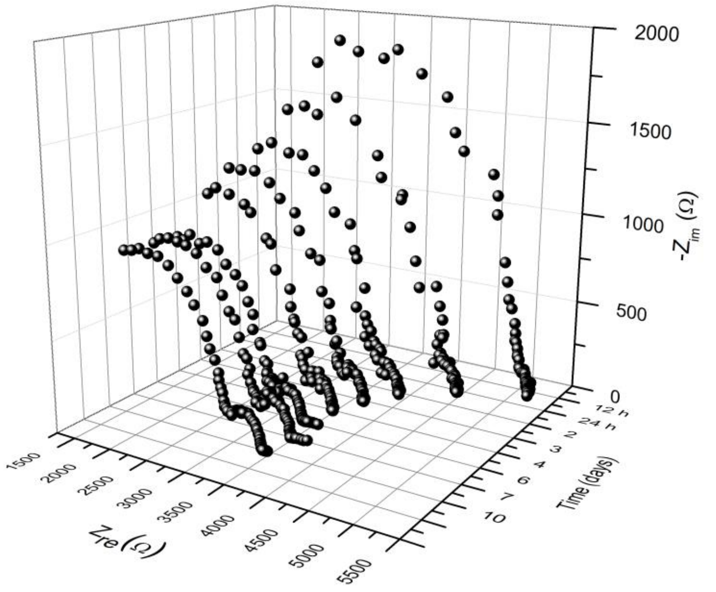

Figure S16. Interfacial stability analysis over time at 70°C of poly[TMC_n-*b*-(LiM_m-*r*-$PEGM_k$)] (**copoly8**) sandwiched in a 2 lithium metal electrodes cell.

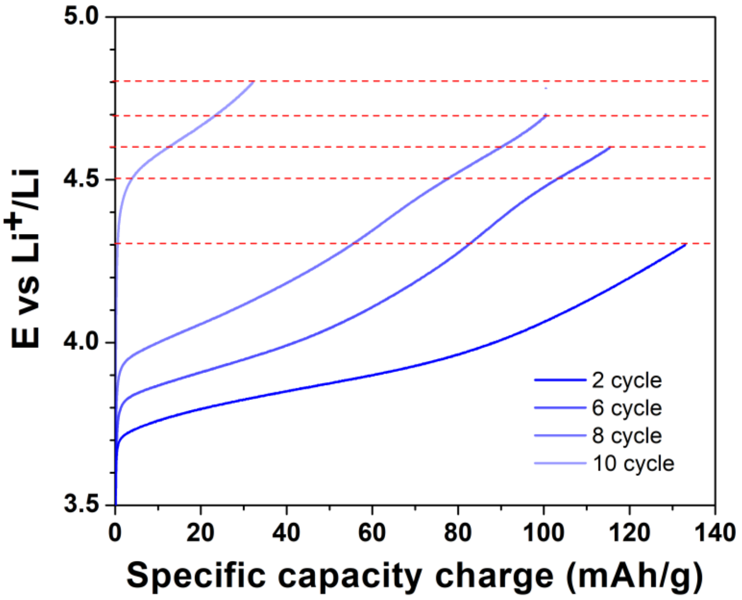

Figure S17. Charge/discharge potential vs specific capacity profiles at C/20 constant current rate from 4.3 to 4.8 V vs. Li^+/Li with constant increment of 0.1 V each 2 cycles for Li/**copoly8**/NMC cell at 70°C.

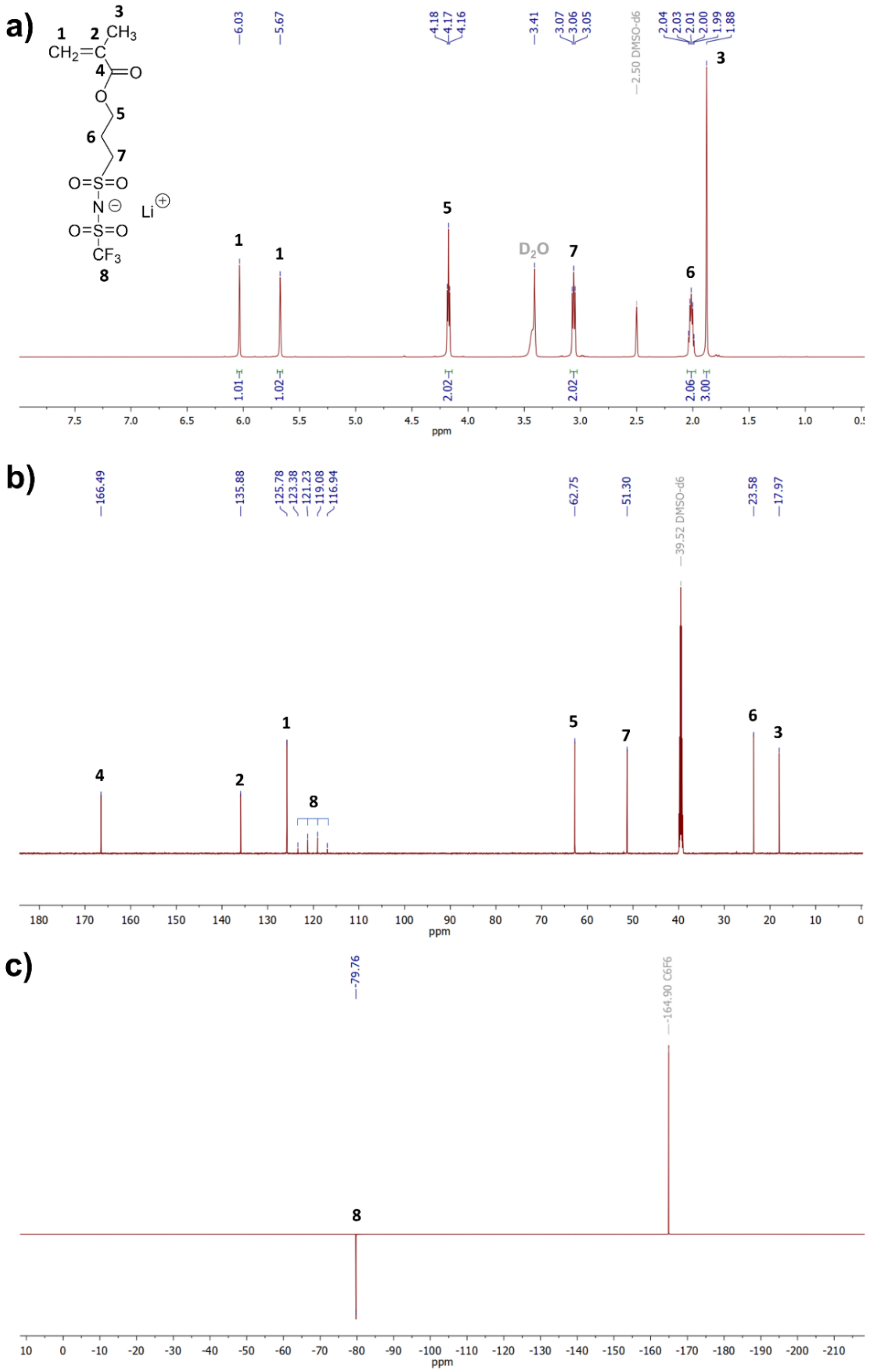

Figure S18. ^{1}H (**a**), ^{13}C (**b**) and ^{19}F NMR (**c**) of LiM monomer (25 °C, DMSO-d_6).

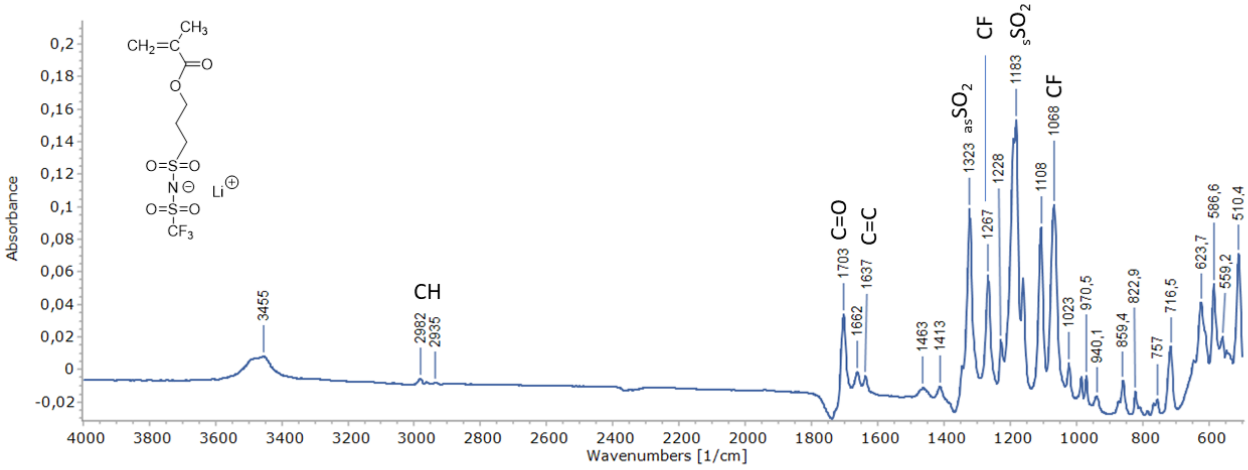

Figure S19. FT-IR spectrum of LiM.

III. SUPPLEMENTARY REFERENCES

(1) Porcarelli, L.; Shaplov, A. S.; Salsamendi, M.; Nair, J. R.; Vygodskii, Y. S.; Mecerreyes, D.; Gerbaldi, C. Single-Ion Block Copoly(Ionic Liquid)s as Electrolytes for All-Solid State Lithium Batteries. *ACS Appl. Mater. Interfaces* **2016**, *8* (16), 10350–10359.

(2) Shaplov, A. S.; Vlasov, P. S.; Armand, M.; Lozinskaya, E. I.; Ponkratov, D. O.; Malyshkina, I. A.; Vidal, F.; Okatova, O. V.; Pavlov, G. M.; Wandrey, C.; Godovikov, I. A.; Vygodskii, Y. S. Design and Synthesis of New Anionic "Polymeric Ionic Liquids" with High Charge Delocalization. *Polym. Chem.* **2011**, *2* (11), 2609–2618.

(3) Abraham, K. M.; Jiang, Z.; Carroll, B. Highly Conductive PEO-like Polymer Electrolytes. *Chem. Mater.* **1997**, *9* (9), 1978–1988.

(4) Evans, J.; Vincent, C. A.; Bruce, P. G. Electrochemical Measurement of Transference Numbers in Polymer Electrolytes. *Polymer (Guildf).* **1987**, *28* (13), 2324–2328.
